# HDAC6-dependent deacetylation of NGF dictates its ubiquitination and maintains primordial follicle dormancy

**DOI:** 10.7150/thno.95164

**Published:** 2024-03-25

**Authors:** Tuo Zhang, Yuntong Tong, Rengguang Zhu, Yaoyun Liang, Jixian Zhang, Chujiao Hu, Meina He, Zhu Hu, Zhiyi Shen, Jin Niu, Jingjing Zhang, Yuanyuan Yu, Bangming Jin, Shan Lei, Zhirui Zeng, Yingmin Wu, Zengmei Cheng, Ziwen Xiao, Bing Guo, Shuyun Zhao, Guoqiang Xu, Wei Pan, Tengxiang Chen

**Affiliations:** 1 Transformation Engineering Research Center of Chronic Disease Diagnosis and Treatment, Department of Physiology, College of Basic Medicine, Guizhou Medical University, Guiyang, Guizhou, 550009, China.; 2 Prenatal Diagnosis Center in Guizhou Province, the Affiliated Hospital of Guizhou Medical University, Guiyang, Guizhou, 550009, China.; 3 Guizhou Provincial Key Laboratory of Pathogenesis & Drug Research on Common Chronic Diseases, Guizhou Medical University, Guiyang, Guizhou, 550009, China.; 4 Reproductive Medicine Center, Department of Obstetrics and Gynecology, The Affiliated Hospital of Guizhou Medical University, Guiyang, Guizhou Province, Guiyang, Guizhou, 550009, China.; 5 Department of Obstetrics and Gynecology, the Affiliated Hospital of Guizhou Medical University, Guiyang, Guizhou, 550009, China.; 6 Guizhou Institute of Precision Medicine, Affiliated Hospital of Guizhou Medical University, Guiyang, Guizhou, 550009, China.; 7 College of Pharmacy, Guizhou Medical University, Guizhou Medical University, Guiyang, Guizhou, 550009, China.

**Keywords:** HDAC6, NGF, ovary, primordial follicle, fertility preservation

## Abstract

**Rationale:** Primordial follicles are limited in number and cannot be regenerated, dormant primordial follicles cannot be reversed once they enter a growth state. Therefore, the length of the female reproductive lifespan depends on the orderly progression and selective activation of primordial follicles, the mechanism of which remains unclear.

**Methods:** We used human ovarian cortical biopsy specimens, granulosa cells from diminished ovarian reserve (DOR) patients, *Hdac6*-overexpressing transgenic mouse model, and RNA sequencing to analyze the crucial roles of histone deacetylase 6 (HDAC6) in fertility preservation and primordial follicle activation.

**Results:** In the present study, we found that HDAC6 was highly expressed in most dormant primordial follicles. The HDAC6 expression was reduced accompanying reproductive senescence in human and mouse ovaries. Overexpression of *Hdac6* delayed the rate of primordial follicle activation, thereby prolonging the mouse reproductive lifespan. Short-term inhibition of HDAC6 promoted primordial follicle activation and follicular development in humans and mice. Mechanism studies revealed that HDAC6 directly interacted with NGF, reducing acetylation modification of NGF and thereby accelerating its ubiquitination degradation. Consequently, the reduced NGF protein level maintained the dormancy of primordial follicles.

**Conclusions:** The physiological significance of the high expression of HDAC6 in most primordial follicles is to reduce NGF expression and prevent primordial follicle activation to maintain female fertility. Reduced HDAC6 expression increases NGF expression in primordial follicles, activating their development and contributing to reproduction. Our study provides a clinical reference value for fertility preservation.

## Introduction

Fertility generally refers to the ability to bring forth offspring, which is the basis for the continuation of populations and evolution of species, as well as the guarantee of human reproduction and social development 1-3. Fertility is showing a decreasing trend worldwide and is facing an unprecedented crisis 4-6. Since the women's fertility after sexual maturity shows an irreversible decline with age, numerous factors affect and even destroy it. Female fertility is usually more specifically referred to as the protection and preservation of ovarian function, especially the quality and quantity of oocytes 7-9. Follicles containing oocytes are also important for female fertility preservation. The majority of follicles in the ovary are primordial follicles, which are dormant to maintain female fertility. The development of dormant primordial follicles into primary follicles and their activation occurs throughout the reproductive cycle 10, 11. Because primordial follicles have a limited and non-renewable reserve, dormant primordial follicles cannot be reversed once they enter a growth state 12. Therefore, selective activation of primordial follicles directly influences the rate of follicle depletion and thus determines the length of the female reproductive life span. Excessive activation of primordial follicles causes unnecessary follicle consumption, premature ovarian failure, and shortened reproductive lifespan. On the contrary, excessive inhibition of primordial follicle activation can also lead to female infertility. Properly controlling the primordial follicle activation prolongs the reproductive life of the ovaries 10, 11.

The primordial follicle consists of a centrally located oocyte surrounded by pre-granulosa cells. The pre-granulosa cells, oocytes, and many factors in the primordial follicle microenvironment regulate primordial follicle activation. This complex process involves a plethora of factors, including oocyte-driven factors, such as phosphatase and tensin homolog (PTEN), forkhead box O3 (FOXO3a), LIM homeobox 8 (LHX8), NOBOX neogenesis homeobox (NOBOX), spermatogenesis and oogenesis-specific basic helix-loop-helix (SOHLH), KIT, cell division cycle 42 (CDC42), and E-cadherin (E-cad) 13-19. Other factors playing essential roles in primordial follicle activation and follicular reserve maintenance include pre-granulosa cell-driven factors, such as KIT Ligand (KITL), mechanistic target of rapamycin kinase (mTOR), forkhead box l2 (FOXL2), SMAD family member 3 (SMAD3) and WNT 20-23. Also, participation of cyclin dependent kinase inhibitor 1B (CDKNIB), HIPPO, nerve growth factor (NGF), transforming growth factor-beta (TGF-β), Rho-associated coiled-coil containing protein kinase 1 (ROCK1), cyclin AMP (cAMP), and Sirtuin 1 (SIRT1) has been reported in primordial follicle activation and reserve maintenance 24-32.

Despite the involvement of several paracrine and autocrine factors in regulating primordial follicle activation and maintenance of primordial follicular reserve, the underlying mechanisms remain unclear. In all follicle developmental stages, the transforming growth factor-beta superfamily is expressed in oocytes, granulosa cells, and interstitial cells 33. TGF-β signaling pathway can regulate the maintenance and activation of the primordial follicle pool by modulating the TSC/mTORC1 signaling pathway 22, 31, 34. The neurotrophin or neuropeptide family is essential for cell survival, differentiation, and development in the nervous system and non-neuronal tissues 35, 36. Neurotrophins and their receptors are involved in mammalian ovarian development 37-41.

Mammalian ovaries, including from humans, mice, rats, goats, cows, and rabbits, express NGF and its high-affinity receptor, neurotrophic tyrosine kinase receptor 1 (TRKA), low-affinity receptor P75NTR (p75), brain-derived neurotrophic factor (BDNF), and neurotrophic tyrosine kinase receptors 2 (TRKB) and 3 (TRKC) 42, 43. Additionally, NGF, TRKA, BDNF, and TRKB play a role in oogenesis, follicular development, oocyte maturation, and early embryo development 30, 37, 44. TRKB agonist antibodies have been reported to improve fertility in naturally aging and chemotherapy-induced premature ovarian failure mice 45. NGF in interstitial cells acts upstream of mTOR to control primordial follicle selective activation 30. The upstream regulatory mechanisms that regulate the expression of TSC, TGF-β, NGF, and BNDF during primordial follicle activation remain unclear.

As a member of the histone deacetylase family, HDAC6 is unique with two deacetylation catalytic domains and one ubiquitination domain, which can promote deacetylation modification and ubiquitination degradation of several protein substrates, including histones and non-histone proteins 46, 47. The first deacetylation domain is primarily responsible for deacetylating cortactin and HSP90, while the second catalytic domain deacetylates α-Tubulin 48, 49. The ubiquitination domain promotes the degradation of misfolded proteins 50, 51.

Our previous study showed that HDAC6 is important in balancing primordial follicle dormancy and activation through the mTOR signaling pathway. HDAC6 is highly expressed in most primordial follicles. Interestingly, HDAC6 is expressed at a low level in about 3-4% of primordial follicles, which are activated. Knockdown of *Hdac6* promotes primordial follicle activation, and its overexpression delays their activation 52. The reproductive lifespan of transgenic mice *Hdac6*-OE was extended; more follicles were observed in 16-month-old *Hdac6*-overexpressing mice than in 12-month-old wild-type mice 53. However, the exact mechanism of how HDAC6 extends reproductive life in mice remains unclear. The rate of primordial follicle activation and the rate of follicle depletion determine the length of ovarian fertility 10.

We hypothesized that overexpression of *Hdac6* prolongs the reproductive lifespan of mice by delaying primordial follicle activation and reducing follicle depletion and is involved in the maintenance of female fertility. In this study, we analyzed the published sequencing data of human and mouse follicles at different developmental stages 54, 55. We found that HDAC6 expression was relatively high in primordial follicles but was significantly decreased in diminished ovarian reserve (DOR) of patients, natural ovarian aging. and chemotherapy-induced premature ovarian failure models in mice. Our results showed that overexpression of *Hdac6* delayed primordial follicle activation, thereby prolonging reproductive lifespan in transgenic mouse models. Short-term inhibition of HDAC6 promoted primordial follicle activation and follicular development in humans and mice.

Further studies revealed that HDAC6 interacted directly with NGF, BDNF, TGF-β, and TSC2. HDAC6 reduced the acetylation level of NGF, which was ubiquitinated and degraded following decreased NGF acetylation. Low levels of NGF did not activate the mTOR signaling pathway, so the primordial follicles remained dormant. The physiological significance of the high expression of HDAC6 in most primordial follicles was to reduce NGF expression and maintain the dormancy of primordial follicles, protecting female fertility. When HDAC6 expression was reduced in a few primordial follicles, NGF expression was increased, activating the growth and development of primordial follicles and contributing to reproductive output. Our study provides clinical guidance for the diagnosis of DOR and fertility preservation.

## Materials and Methods

### Animals

Adult CD-1 mice were purchased from Changsha Tianqin Biotechnology Co. Immunodeficient M-NSG female mice were provided by Shanghai Model Organisms Center, Inc. Adult C3H/HeJ (C3H) mice and C57BL/6J (C57) mice were purchased from SpePharm (Beijing, China) Biotechnology Co., Ltd. All mice were housed under controlled lighting (12 h light, 12 h dark) and temperature (24 °C-26 °C) conditions with free access to food and water. Females in estrus were selected at 5 p.m. to be combined with adult males in a 1:1-3:1 ratio, and vaginal plugs were checked at 8 a.m. the next morning. Vaginal plug detection was considered as 0.5-day post coitus (dpc). The first 12 hours after birth was considered 0 days post-partum (dpp). All procedures were conducted in accordance with the guidelines of and approved by the Animal Research Committee of Guizhou Medical University (NO2100006).

### Cisplatin-induced premature ovarian failure mouse model

Adult female 6-8 weeks CD-1 mice were given a daily intraperitoneal injection of 2 mg/kg of cisplatin (S1166, Selleck, USA) 56. The control group mice were injected with the same volume of saline solution. The ovarian tissue was harvested after 14 days.

### Natural aging mouse model

Adult female 6-8 weeks CD-1 mice of around 25 g were selected and housed until 12 months 45. Then ovarian tissues were collected. Female mice were not caged with male mice during housing to minimize individual differences.

### *Hdac6*-overexpressing transgenic mouse (*Hdac6*-OE mouse)

*Hdac6*-overexpressing transgenic mice were provided by Cyagen Biosciences (Suzhou, China) Inc (Agreement Number: KICMRS220803SC1). The gRNA to mouse ROSA26 gene, the donor vector containing "CAG promoter-Kozak-Mouse *Hdac6* CDS-3xEAAAK-EGFP-rBG pA" cassette, and Cas9 mRNA were co-injected into C57BL/6J fertilized mouse eggs to generate targeted knock-in offspring, as illustrated in Figure 2A. F0 founder animals were identified by PCR followed by sequence analysis, shown in Figure 2B, and were bred to wild-type mice to test germline transmission and F1 animal generation. The primers for genotype identification of mice were as follows:

PCR primers for wild-type genotype (annealing temperature 60.0 °C, wildtype allele PCR product: 453 bp): WT-F: CACTTGCTCTCCCAAAGTCGCTC; WT-R: ATACTCCGAGGCGGATCACAA-3; PCR Primers for mutant genotype (annealing temperature 60.0 °C, mutant allele PCR product: 253 bp): Mutant-F: TGAGCAAAGACCCCAACGAGAAG; Mutant-R: CTTTATTAGCCAGAAGTCAGATGC; Homozygous: one band of 253 bp; Heterozygous: two bands of 253 and 453 bp; WT: one band of 453 bp.

### *In vivo* live imaging of *Hdac6*-OE mouse

The green fluorescence protein (GFP) expression in the *Hdac6*-OE mouse was observed using the small animal *in vivo* optical two-dimensional imaging system (IVIS Lumina Ⅲ, perkinelmer, USA) according to the manufacturer's instructions.

### Isolation of newborn mouse ovaries

Newborn mouse ovaries were isolated using a previously reported method 26. Briefly, the surfaces of the newborn female mice were sterilized with 75% alcohol, and mice were sacrificed by cervical dislocation. The abdomen was dissected with ophthalmic scissors to expose the kidney, and the ovary was located below the kidney. The mouse body was cut along the anterior end of the kidney with ophthalmic scissors, leaving the kidney and ovary attached to the hind limb on the lower half of the mouse body. The lower half of the carapace was placed in a sterile ice-cold PBS solution. Then, the mouse ovary, which was encased by the ovarian capsule, was separated by ophthalmic forceps under a body microscope and placed in another sterile ice-cold PBS solution. The ovarian capsule was peeled off with an 1 mL syringe under a microscope, leaving bare the ovarian tissue. Damage to the ovary was avoided during the surgical procedure, which can lead to follicular overgrowth 30, 57.

### Ovarian organ culture

The ovarian culture procedure was according to previous reports 20, 26, 58. Briefly, 1.2-1.4 mL of Dulbecco's Modified Eagle's Medium (DMEM) medium was added to a 6-well plate with low adsorption, placed in a cell culture incubator, and equilibrated for 15-20 min. A 20-50 μL pipette was used to transfer 6-10 newborn mouse ovaries to each well of the pre-warmed 6-well plate which were cultured in suspension. The ovaries were cultured with 4 μM Tubastatin A (TubA) (S8049, Selleck, USA), 30 nM K252a (HY-N6732, MedChemExpress, USA), 20 μM ANA-12 (S7745, Selleck, USA) at 37 °C with 5% CO_2_. The medium was changed every other day.

### Gene knockdown in newborn mouse ovaries

The *Hdac6* knockdown vectors were mixed with 4% trypan blue solution (15250061, Gibco, USA) (vector/trypan blue solution: 10/1). The mixture was injected into isolated 2 dpp mouse ovaries using glass pipettes under a stereomicroscope. Electrotransfection was performed by applying three 5-ms-long quasi-square pulses at a pulse-field strength of up to 30 V/cm according to previous reports 25, 52. The *Hdac6* knockdown vector was constructed by cloning *Hdac6*-shRNA, GGTACTTCCCATCGCCTATGA.

### Cell culture

KGN cells were a gift by Professor Chao Wang from China Agricultural University. KGN cells were cultured in DMEM supplemented with 10% fetal bovine serum (04-001-1A, Biological Industries) and 1% penicillin/streptomycin (P1400, Solarbio). Cell lines were cultured at 37 °C with 5% CO_2_. The absence of mycoplasma contamination was ascertained in cultured cells using the Mycoplasma Stain Assay Kit (C0296, Beyotime, China). Lipofectamine 3000 (L3000075, ThermoFisher, USA) was used to transfect the knockdown or overexpression plasmid vector into cells following the instruction manual. The overexpression plasmids of the human HDAC6 gene, HDAC6-OE, HDAC6-M2, HDAC6-N503 and HDAC6-N840, were obtained from HonorGene (Changsha, China).

### Kidney capsule transplantation

Kidney dorsal membrane transplantation in immunodeficient M-NSG female mice was performed following the reported methods 18, 26. Briefly, immunodeficient mice were anesthetized with sodium pentobarbital, and the dorsal skin was thoroughly disinfected after the hair on the back of the mice was clipped. Longitudinal incisions of approximately 1-1.5 cm were made in the skin and muscle layers from both sides of the spine, where the kidneys were located, using a scalpel. The kidneys were pulled out of the body through the incision by gently squeezing with the fingers, and a 1-mm incision was scratched with the tip of an insulin syringe to separate the kidney from the dorsal membrane in the area where the ovary was to be transplantedusing a blunt-tipped capillary elbow. The ovary was then pipetted and placed in the opening of the dorsal membrane of the kidney with a blunt-tipped capillary elbow. The dorsal membrane of the kidney was then scalded with a hot insulin syringe needle to seal the opening. One kidney was transplanted with control ovaries and the other was transplanted with TubA group ovaries. The ovarian tissues were harvested 14 days after transplantation for subsequent experiments as illustrated in Figure 3.

### Ovarian section immunofluorescence

Fresh ovarian tissues were collected and placed in 1.5 mL centrifuge tubes, and 1 mL of 4% paraformaldehyde was added to fix ovaries for 8-24 h, 8 h for newborn ovaries, and 24 h for adult ovaries. Gradient alcohol dehydration was performedwith the dehydration time proportional to the size of the ovarian tissue, usually 5-20 min. Tissue clearing was performed using xylene, typically 5-20 min. Tissue dipping in the wax was performed for 2-8 h, 2 h for newborn mouse ovaries and 8 h for adult ovaries. After wax dipping, tissues were sectioned at 5 μm thickness for ovaries less than 4 days old and 8 μm for ovaries greater than 4 days old. Tissue sections were baked overnight in an oven at 42 °C until the ovarian tissues were tightly adhered to the slides and subjected to xylene dewaxing and gradient alcohol rehydration. The rehydrated sections were washed twice with PBS for 5 min each time. High-pressure antigen repair with citrate buffer was then performed for 20 min. Slices were kept at room temperature to cool naturally followed by 2 washes with PBS at 100 rpm for 5 min each. Subsequently, the sections were treated with 0.1% Triton X-100 (85111, Thermo Scientific, USA) for 15-20 min followed by 2 washes with PBS at 100 rpm for 5 min each, and blocked with 5% BSA for 1-2 h at room temperature. BSA was then removed and tissue sections were incubated with the primary antibody at 4 °C for 12-48 h. The antibodies used are listed in the Supplementary Table 1. Next, the sections were washed 3 times with PBS at 100 rpm for 10-20 min each time, then incubated with the fluorescent secondary antibody for 1 hour at 37 °C protected from light. Subsequently, tissue slices were washed twice with PBS at 100 rpm for 5 min each time. Finally, the ovarian sections were blocked with anti-fluorescence quenching sealer (20230616; RuiTaibio, China) and photographed with an Olympus confocal microscope. Pictures of the control and treatment groups were not altered for intensity and/or contrast.

### Cell immunofluorescence

KGN cells were cultured with or without 4 μM TubA for 24 h, washed twice with PBS, fixed with 4% paraformaldehyde for 4-8 h at 4 °C, and permeabilized with 0.1% Triton X-100 in PBS for 30-45 min. Subsequently, cells were incubated with the primary antibody for 12-48 h at 4 °C. The primary antibodies were recycled for use no more than three times. Cells were washed twice with PBS at 100 rpm for 15 min. The secondary antibody was then added to the cells and incubated for 1 h at 37 °C followed by 3 washes with PBS for 15 min. Nuclei were stained with DAPI (C1005, Beyotime, China). The cells were again washed 2 times for 5 min and then photographed with the Olympus confocal microscope. Pictures of the control and treatment groups were not altered for intensity and/or contrast. The antibodies used are listed in Supplementary Table 1.

### Quantification of ovarian follicles

Ovarian follicles were counted using established procedures 59. Briefly, ovarian sections before 4 dpp were cut at 5 μm, and those after 4 dpp were cut at 8 μm. The sections were stained with hematoxylin, and follicles with clear oocyte nuclei were counted. Follicles at different stages of development were categorized as primordial, primary, secondary, and antral follicles according to the reported method.

### Quantification of granulosa cell thickness

The quantification of granulosa cell thickness was performed following the reported methods 23, 60. Briefly, the sections were stained with hematoxylin, follicles with clear oocyte nuclei were measured the granulosa cell thickness with photoshop software.

### Western blotting analysis

Ovarian protein samples or cell protein samples were extracted in the WIP tissue lysis buffer containing phenylmethylsulfonyl fluoride (8553S, Cell Signaling Technologies, USA) or RIPA lysis buffer (P0013B, Beyotime, China) according to the manufacturers' instructions. The protein concentration was assessed by a BCA protein assay kit (P0012, Beyotime, China). Protein samples were separated by 10%-15% SDS-PAGE and then transferred to polyvinylidene fluoride membranes (IPVH00010, Millipore, USA). The membranes were blocked with 5% skim milk powder for 1 h at room temperature and then incubated with primary antibodies for 12-48 h at 4 °C. The primary antibodies used are listed in Supplementary Table 1. The membrane was washed three times with TBST for 10 min each time. The membranes were incubated with the secondary antibody for 1 h at room temperature and washed three times with TBST for 10 min each time. The secondary antibodies were horseradish peroxidase-conjugated goat anti-mouse IgG (H+L) (ZB-2305, Zhongshan Golden Bridge, China) and horseradish peroxidase-conjugated goat anti-rabbit IgG (H+L) (ZB-5301, Zhongshan Golden Bridge, China) diluted 1:10000. The membranes were visualized using a SuperSignal West Pico Chemiluminescent Detection System (5200, Tanon, China). β-actin was used as the intrinsic control.

### Co-immunoprecipitation (Co-IP) assay

The kidney, liver, and ovary tissues from 7 dpp wild-type and *Hdac6*-OE mice were collected for Co-IP experiments. Protein extraction and quantification were carried out with the protocol used for Western blotting. About 5%-10% tissue proteins were saved as input analysis, and 2 mg of protein was loaded onto pre-washed protein A/G magnetic beads for 30 min at 4 °C. Tissue lysates were immunoprecipitated with the indicated antibodies for 18 h at 4 °C. Next, the protein A/G magnetic beads were added and incubated for 2 h at 4 °C to recover the IP complexes. The beads were washed five times with NP-40 buffer using a magnetic separator. The bound proteins were eluted with 2×SDS buffer. The proteins were detected according to the protocol used for Western blotting.

### Real-Time PCR analysis

Total RNA was extracted with TRIzol (15596026, ThermoFisher, USA) and cDNA was synthesized with the high-capacity cDNA reverse transcription kit (15596026, ThermoFisher, USA) according to the manufacturers' protocols. Real-Time PCR analysis was performed using SYBR Select Master Mix and the Bio-Rad CFX96 Real-Time PCR System. The data were normalized to *β-actin*. The primers used are listed in Supplementary Table 1.

### RNA-seq and data analysis

The 2 dpp CD-1 female mouse ovaries cultured with TubA for 2 days were collected to extract total RNA as described above. RNA-seq was performed by LC-Bio Technology Co., Ltd. (Hangzhou, China). Differentially expressed genes (DEGs) were identified between the two groups at a *P* value of <5% and absolute log_2_ Fold Change ≥ 1. The differentially expressed genes are presented in supplemental table 2. Gene Ontology (GO) and Kyoto Encyclopedia of Genes and Genomes (KEGG) pathway enrichment analyses were performed to determine the functions of the differentially expressed genes. OmicStudio tools at https://www.omicstudio.cn/tool were used for bioinformatic analysis. Gene set enrichment analysis (GSEA) was performed using GSEA software.

### Collection of human ovarian tissue and human granulosa cells

Six women aged 31-35 years donated small ovarian cortical biopsy specimens (adjacent non-tumor tissue) while undergoing routine gynecological laparoscopies at the Reproductive Medicine Center, the Affiliated Hospital of Guizhou Medical University, Guiyang, China. Written informed consents were obtained before surgery. The isolation and purification of human granulosa cells from human ovarian follicular fluid were performed according to the reported method 61. Granulosa cells from patients with diminished ovarian reserve (DOR) were collected to detect changes in HDAC6. Granulosa cells from patients whose husbands were infertile served as controls. This study was performed in accordance with the Declaration of Helsinki. Approval for this study was obtained from the Ethical Committee of the Affiliated Hospital of Guizhou Medical University (No. 202148).

### Protein molecular docking

The binding modes between the proteins were predicted from their protein structures (HDAC6 PDB:6UO2 BDNF PDB:1B8M 1VJF NGF PDB: 1SGF) obtained from the PDB database (https://www.rcsb.org/). No analyzed structure exists for TSC2, so, its protein model was predicted using AIphaFold (https://alphafold.com/). HDAC6 was then docked with NGF, BDNF, TGF-beta, and TSC2using the ZDOCK (https://zdock.umassmed.edu/) protein-protein online platform. The optimal model from the docking results was further analyzed in PDBePISA (https://www.ebi.ac.uk/msd-srv/prot_int/pistart.html) to determine the interaction interfaces and bonding relationships, and finally completed in Pymol mapping.

### Statistical analyses

Statistical analyses were carried out using GraphPad Prism 8.0. Data were expressed as the mean of at least three independent experiments. The results were presented as means ± SDs. Two-tailed unpaired Student's t-test and one-way analysis of variance followed by Tukey's post-hoc test were used to analyze the statistical significance between two groups and among multiple groups, respectively. The statistical significance was set at *P* value < 0.05.

## Results

### HDAC6 expression is decreased during ovarian aging

**Figure 1:**
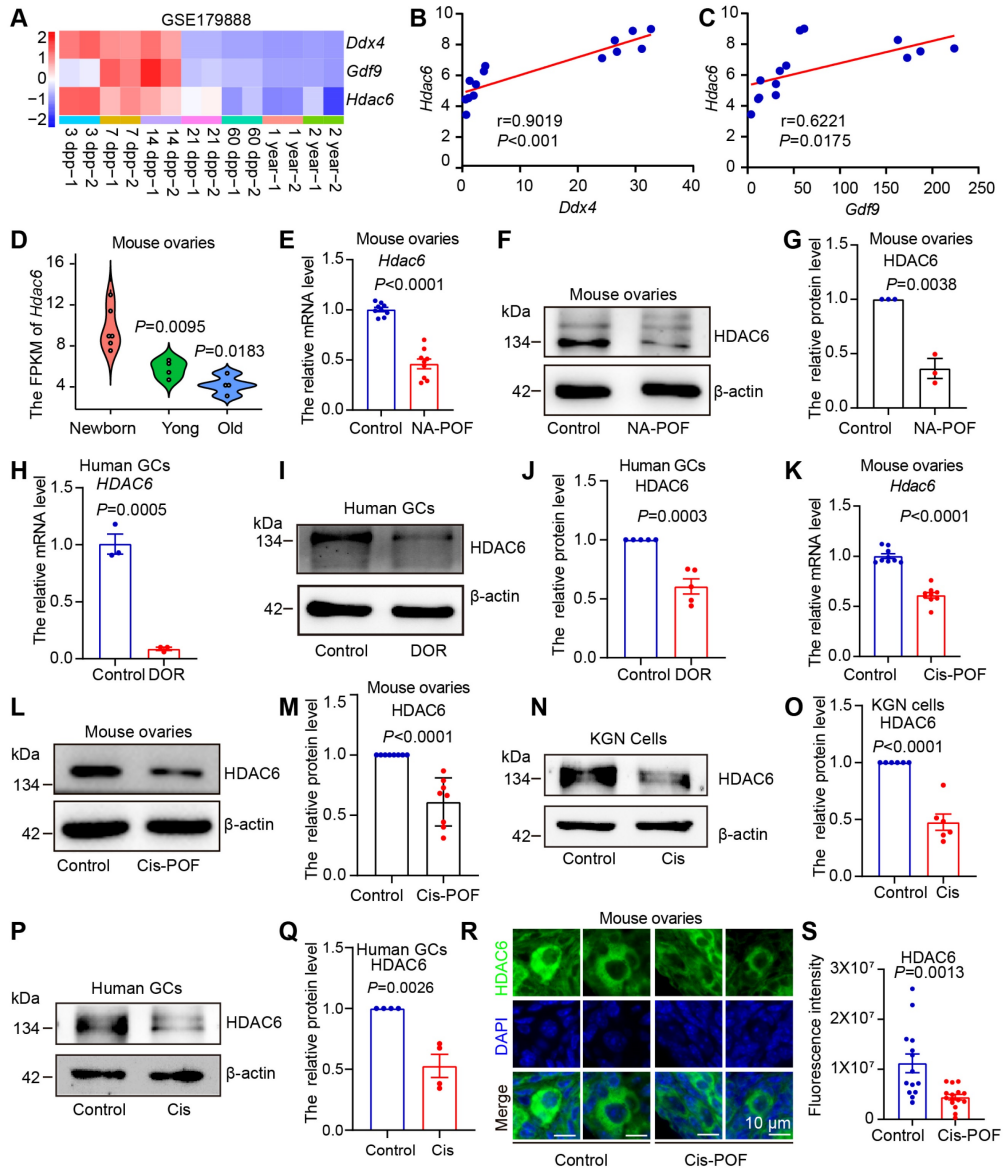
** Expression of HDAC6 is decreased during natural and chemotherapeutic ovarian aging in mice and humans. (A)** Analysis of *Hdac6*, *Gdf9,* and *Ddx4* expression during mouse ovarian development and aging by transcriptome sequencing from ovarian tissues at different time points (GSE179888). **(B)** Correlation analysis of *Hdac6* and *Ddx4* expression during mouse ovarian development and aging (GSE179888). **(C)** Correlation analysis of *Hdac6* and *Gdf9* expression during mouse ovarian development and aging (GSE179888). **(D)** Expression of *Hdac6* in newborn mouse ovaries, young mouse ovaries, and old mouse ovaries (GSE179888). **(E)** mRNA levels of *Hdac6* in young mouse ovaries and naturally aging mouse ovaries. **(F)** Total protein level of HDAC6 in young mouse ovaries and naturally aging mouse ovaries. **(G)** Statistical analysis of Figure F. n=3. **(H)** *HDAC6* mRNA levels in ovarian granulosa cells from normal individual and DOR patients. **(I)** HDAC6 protein level in ovarian granulosa cells from normal individual and DOR patients. **(J)** Statistical analysis of Figure I. n=5. **(K)** *Hdac6* mRNA levels in young mouse ovaries and cisplatin-induced ovarian aging. **(L)** HDAC6 protein levels in young mouse ovaries and cisplatin-induced ovarian aging. **(M)** Statistical analysis of Figure L. n=8. **(N)** HDAC6 protein level in cisplatin-treated KGN cells for 48 h. **(O)** Statistical analysis of Figure M. n=6. **(P)** HDAC6 protein level in cisplatin-treated human primary granulosa cells. **(Q)** Statistical analysis of Figure P. n=4. **(R)** Immunofluorescent staining of HDAC6 (green) in young ovaries and cisplatin-induced aging ovaries. The nuclei were stained with DAPI (blue). Scale bars, 5 μm. **(S)** Fluorescence intensity analysis in primordial follicles from cisplatin-induced aging ovaries.

We explored the potential function of HDAC6 during ovarian development and aging by first analyzing published transcriptome sequencing data from 3, 7, 14, 21, 60 dpp, 1-year, and 2-year mouse ovaries 55. Specific expression of DDX4 and GDF9 were reported in the cytoplasm of oocytes 62, 63, whereas NOBOX and Figlα were expressed in the nucleus of oocytes 16. AMHR2 is the type II receptor for AMH, secreted by granulosa cells of small, growing follicles, and is an important clinical indicator of ovarian reserve 64. In mice, the primordial follicular pool is established 3-7 days after birth, and some follicular loss occurs in puberty (21 days) 10, 65. A relatively high expression level of *Ddx4*, *Gdf9*, *Nobox*, *Figlα*, and *AMHR2* was maintained until day 21 and then decreased when the ovarian follicles were gradually lost after entering day 60. (Figure 1A, Figure S1A). These changes are consistent with the available information on ovarian development and aging 1. We observed the highest expression of HDAC6 during the high ovarian reserve stage, which is the period with the highest number of primordial follicles (3-7 dpp). HDAC6 remained high during ovarian development, declining dramatically after ovarian aging (Figure 1A, Figure S1A).

Correlation analysis showed that the expression trends of HDAC6 were positively correlated with those of DDX4, GDF9, NOBOX, Figlα, and AMHR2 (Figure 1B-C, Figure S1B-D). We classified 3, 7, and 14 days after birth as the newborn stage and a period of high ovarian reserve, 21 days as the young stage of middle ovarian reserve, and 1-2 years as the old stage of aging ovarian reserve. The expression of *HDAC6* showed a gradual decreasing trend from the newborn to the old stage (Figure 1D).

We analyzed single-cell transcriptome sequencing data from human ovarian granulosa cells and oocytes at different developmental stages 54. In human oocytes, *HDAC6* expression was relatively high in primordial and primary follicle oocytes and low in secondary, luminal, and preovulatory follicles (Figure S1E). In human granulosa cells, *HDAC6* expression was relatively high in primordial, primary. and preovulatory follicles (Figure S1F). We analyzed the transcriptome sequencing data of human granulosa cells from primordial and primary follicles isolated by laser capture microdissection 66. The results showed that the *HDAC6* expression decreased from primordial to primary follicles (Figure S1G). These sequencing data suggested high *HDAC6* expression in ovaries withmany primordial follicles that decreased during ovarian aging. We further utilized a naturally aging mouse model to detect HDAC6 expression 45. HE staining showed that follicles were largely depleted in the ovaries of naturally aging 12-month-old mice (Figure S2A). qPCR and Western blot analyses showed significantly decreased HDAC6 expression in the ovaries of naturally aging aged mice compared with the young mice (6-8 weeks) (Figure 1E-G).

We also collected clinical samples from diminished ovarian reserve (DOR) patients and analyzed the serum sex hormone levels. The results showed that DOR patients had decreased anti-Müllerian hormone (AMH) and estrogen (E2) and increased follicle-stimulating hormone (FSH). Luteinizing hormone (LH), prolactin (PRL), progesterone (PROG), and testosterone (TEST) were unchanged (Figure S2G-M). The results showed that HDAC6 mRNA protein levels were significantly decreased in granulosa cells from DOR patients compared with normal individuals (Figure 1 H-J).

Cisplatin exposure causes hyperactivation or atresia of primordial follicles, thereby triggering premature ovarian failure 56, 67. We injected mice with 2 mg/kg of cisplatin daily and collected ovarian tissues after 14 days 56. HE staining showed that mouse ovaries were significantly smaller in the cisplatin group (Figure S2B). The number of total, primordial, and growing follicles was decreased, and the number of atresia follicles was increased (Figure S2C-F), indicating that the model was of premature ovarian failure. The qPCR and Western blotting results showed a significant decrease in mRNA and protein levels of HDAC6 in cisplatin-treated mouse ovaries (Figure 1K-M). We added cisplatin to cultured KGN cells, the human ovarian granulosa-like tumor cell line, and human primary granulosa cells. A significant decrease in HDAC6 in the KGN cells and human primary granulosa cells was observed after culturing with cisplatin (Figure 1N-Q). Because ovarian reserve mainly refers to primordial follicle numbers 10, we examined HDAC6 expression in cisplatin-treated mouse ovaries. The immunofluorescence results showed that the expression of HDAC6 was significantly decreased in primordial follicles from the cisplatin-treated group (Figure 1 R-S). These results confirmed decreased HDAC6 expression during ovarian aging and suggested that HDAC6 in primordial follicles may play a key role in maintaining ovarian reserve. Therefore, we explored the function and mechanism of HDAC6 in preserving the primordial follicle pool.

### Overexpression of *Hdac6* delays primordial follicle activation in mice

**Figure 2:**
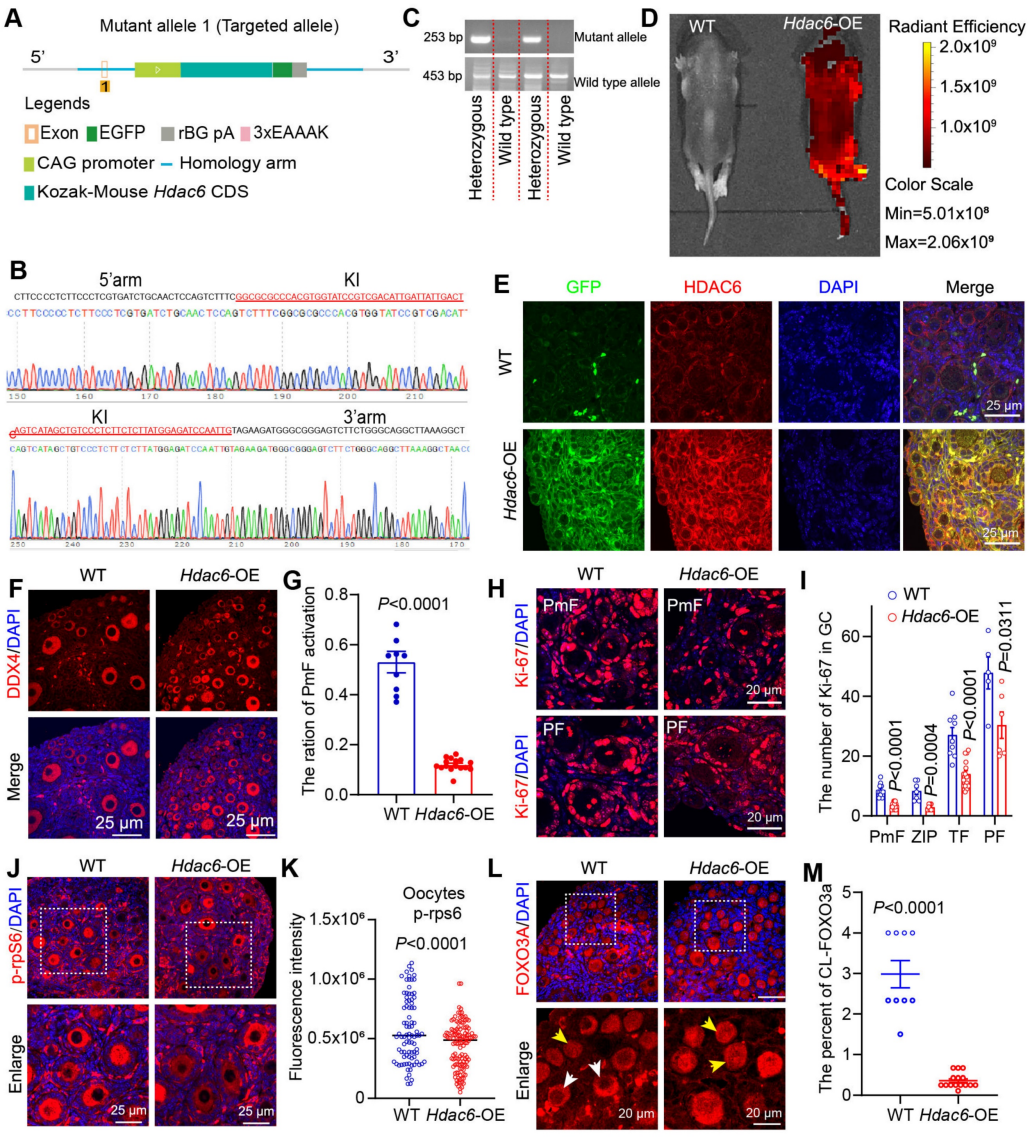
** Overexpression of *Hdac6* delays primordial follicle activation. (A)** Diagram of the establishment of the *Hdac6*-OE transgenic mouse model. **(B)** Knock-in (KI) of exogenous sequence.s **(C)** Genotypes of the transgenic mouse identified by PCR. **(D)** Expression of GFP fusion tags in the entire body of newborn 7-day-old mouse by small animal live imaging.** (E)** Immunofluorescence analysis of *Hdac6* overexpression in the ovaries of 7 dpp *Hdac6*-OE transgenic mouse(red) co-stained with GFP (green). The nuclei were stained with DAPI (blue). Scale bars, 25 μm. **(F)** Immunofluorescence staining of ovary sections from 7 dpp *Hdac6*-OE mice. DDX4 signal (red) as the oocyte marker; nuclei were stained with DAPI (blue). Scale bars, 25 μm. **(G)** Ratio of primordial follicle activation. **(H)** Ki-67 (red) examined by immunofluorescence in 7 dpp *Hdac6*-OE mouse ovarian sections; nuclei were stained by DAPI (blue), scale bars, 25 μm. **(I)** Statistical analysis of Ki-67 positive granulosa cells from PmF, ZIP, TF, and PF in *Hdac6*-OE mouse ovaries. **(J)** p-rpS6 expression level (red) examined by immunofluorescence in 7 dpp *Hdac6*-OE mouse ovarian sections; The nuclei were stained by DAPI (blue), scale bars, 25 μm. **(K)** Statistical analysis of p-rpS6 fluorescence intensity in *Hdac6*-OE mouse oocytes. **(L)** The immunofluorescence of FOXO3a (red) in 7 dpp *Hdac6*-OE mouse ovarian sections; nuclei were stained by DAPI (blue); Scale bars, 25 μm. **(M)** Statistical analysis of the CL-FOXO3a in *Hdac6*-OE mouse ovaries.

Overexpression of *Hdac6* extended the reproductive lifespan of mice, and *Hdac6-*overexpressing mice had more primordial follicles and primary follicles in their ovaries than the control group at 16 months 53. Our previous study showed that overexpression of *Hdac6* delayed primordial follicle activation from newborn mouse ovaries in the *in vitro* culture model 52. We hypothesized that HDAC6 extends the reproductive lifespan of mice by delaying primordial follicle activation. We constructed CAG promoter-driven *Hdac6*-expressing, GFP-tagged transgenic mice (Figure 2A). Sequencing results indicated that the exogenous fragment was indeed knocked into the target region (Figure 2B). PCR analysis showed location of the wild-type band at 453 bp and the mutant band at 253 bp (Figure 2C). Only wild-type band mice were named wild-type (WT), and heterozygous mice with wild-type and mutant bands were named *Hdac6*-OE. Small animal live imaging showed that the GFP fluorescent tag was expressed throughout the body (Figure 2D). The qPCR (Figure S3A-B) and Western blotting (Figure S3C-H) results showed a significant increase in the mRNA and protein levels of *Hdac6* and *Gfp* in the ovaries, heart, liver, spleen, lungs, and kidneys of *Hdac6*-OE mice compared with WT mice.

HDAC6 has been shown to promote α-tubulin (K40) (ac-Tubulin) acetylation levels 68. The protein levels of ac-Tubulin were significantly decreased in ovaries, kidneys, lungs, and liver in *Hdac6*-OE mice (Figure S3C-H). Ovarian tissues were harvested for immunofluorescence assay. The GFP fluorescence signal was barely detectable in the ovaries of wild-type mice, where the green fluorescent dots in Figure 2E may be the autofluorescence of erythrocytes. GFP fluorescence signals were detected in all cells throughout the ovary from *Hdac6*-OE mice (Figure 2E). We used immunofluorescence to detect the expression level of HDAC6 and found it to be significantly upregulated in the oocytes, granulosa cells, and interstitial cell from *Hdac6*-OE mouse ovaries (Figure 2E, Figure S3I). The fluorescence intensity of ac-Tubulin was significantly reduced in the ovaries of *Hdac6*-OE mice (Figure S3J-K). These results demonstrated the successful construction of *Hdac6*-overexpressing mice.

It has been previously reported that a few primordial follicles were activated at 7 dpp 11. Immunofluorescence results in our study showed an increased number of DDX4 positive cells in 7 dpp *Hdac6*-OE mouse ovaries (Figure 2F). Statistical analysis of HE staining showed a significant reduction in the number of primordial and total follicles, primary follicles, and the percentage of primordial follicle activation in *Hdac6*-OE mice ovaries (Figure 2F-G, Figure S4A-D), suggesting that *Hdac6* overexpression increased primordial follicle pool and delayed their activation.

The developmental stage from primordial to primary follicle can be divided into primordial follicle (PmF), zipper-like follicle (ZIP), transitional follicle (TF), and primary follicle (PF) based on the morphology 34. We counted the number of Ki-67-positive granulosa cells of these four types of follicles, and the results showed that their number increased significantly during the development from PmF to PF, especially from ZIP to PF (Figure 2H, Figure S4E). When *Hdac6* was overexpressed, the number of Ki-67 positive cells in these four follicles was significantly reduced (Figure 2I), and the rate of increase in the number of Ki-67-positive cells during primordial follicle activation was reduced (Figure S4F). During primordial follicle activation, phosphorylation of ribosomal protein S6 (p-rpS6) was expressed in interstitial cells, primary follicle granulosa cells, and even more so in primary follicle oocytes (Figure S4G). Also, FOXO3a translocation was was observed from the oocyte nucleus to the cytoplasm 20, 31. Immunofluorescence results showed that the number of p-rpS6 positive oocytes was reduced in the *Hdac6-*OE mouse ovaries. The fluorescence intensity of p-rpS6 was also reduced in oocytes, granulosa cells, and interstitial cells from *Hdac6*-OE mouse ovaries (Figure 2J-K, Figure S4H). There was less cytoplasmic localization of FOXO3a in *Hdac6*-OE mouse ovaries than in WT mouse ovaries (Figure 2L-M). These results suggested that overexpression of *Hdac6* increased the primordial follicle pool and delayed primordial follicle activation.

### Short-term inhibition of HDAC6 promotes primordial follicle activation and follicular development in mouse and human

**Figure 3:**
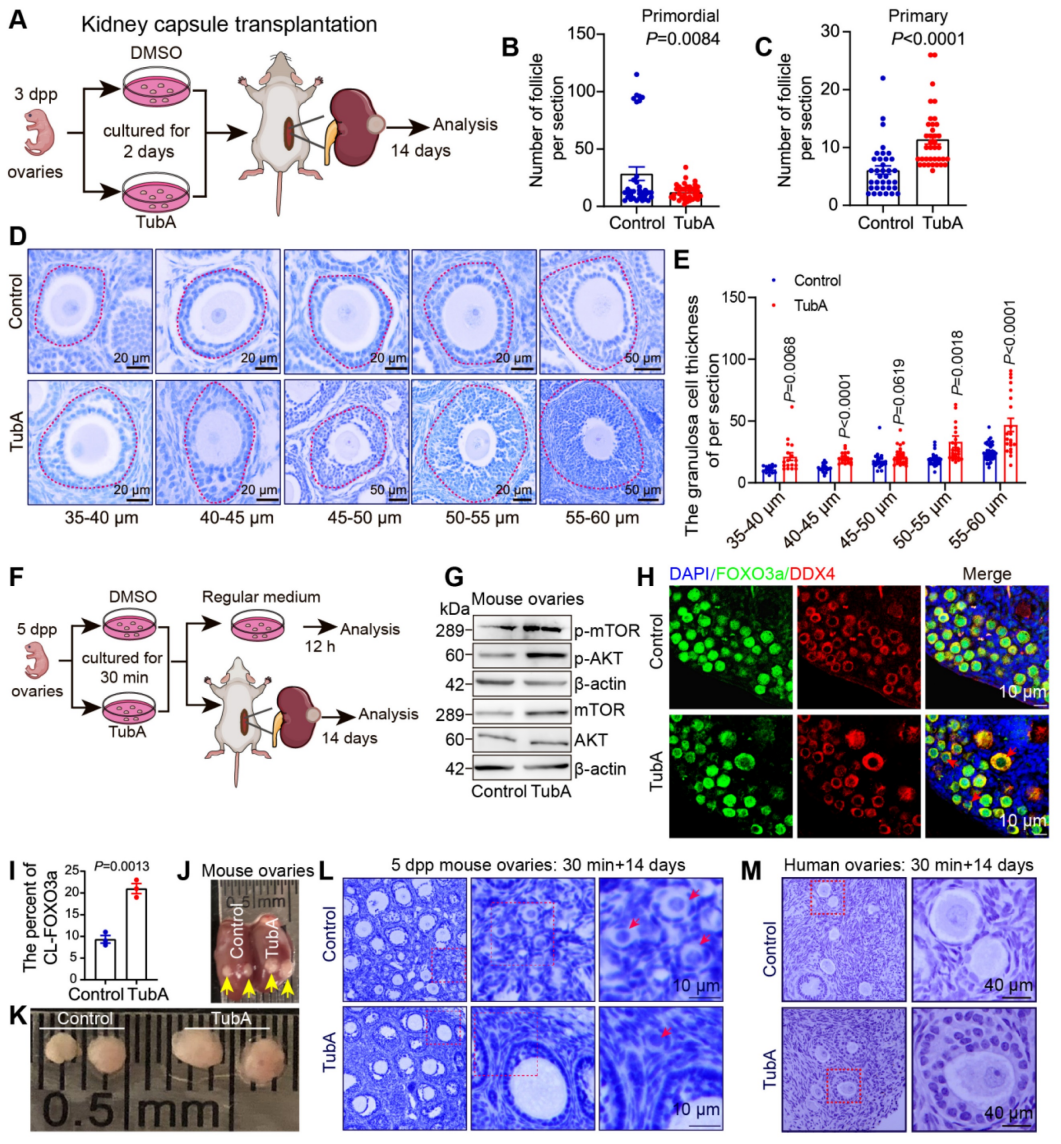
** Suppression of HDAC6 promotes primordial follicle activation and follicular development. (A)** Experimental design for kidney capsule transplantation of TubA-treated ovaries. **(B-C)** Statical results of PmF and PF counts in (A). **(D)** HE staining of ovarian sections from TubA-treated ovaries in (A). **(E)** Quantitation of granulosa cell thickness from oocytes diameters of 35-40, 40-45, 45-50, 50-55, and 55-60 μm. **(F)** Experimental design for kidney capsule transplantation of TubA shotr time treated ovaries. **(G)** Protein levels of AKT, mTOR, p-AKT, and p-mTOR in TubA-treated. 5 dpp ovaries were incubated with TubA for 30 min and then transferred to the blank medium for 12 h. **(H)** Immunofluorescent staining of FOXO3a in TubA-treated ovaries about Figure F. FOXO3a signals (green) were co-stained with DDX4 (red). Nuclei were stained with DAPI (blue). Red arrows indicate CL-FOXO3a. 5dpp ovaries were incubated for 30 min with TubA and then transferred to the regular medium for 12 h. **(I)** Statistical analysis of CL-FOXO3a ratios in Figure H. **(J-K)** Ovarian tissue 14 days after kidney capsule transplantation. Yellow arrows indicate ovarian tissue under the kidney capsule. **(L)** Hematoxylin staining of mouse ovarian sections from TubA-treated ovaries in Figure K. **(M)** Hematoxylin staining of human ovarian sections from TubA-treated ovaries. Human ovarian cortex tissue slices were incubated for 30 min with TubA and then transplanted under the kidney capsule of immunodeficient mice to continue development for 14 days.

Our previous study showed that inhibition of HDAC6 promotes mouse primordial follicle activation in an *in vitro* ovarian culture model 52. However, it was not clear whether primordial follicles could develop normally after activation. We cultured 3 dpp mouse ovaries with TubA for 2 days and then transplanted them below the kidney dorsal membrane in immunodeficient mice for 14 days (Figure 3A). Immunofluorescence and Western blotting showed that the level of ac-Tubulin was decreased in TubA-treated ovaries for 2 days. A reduced number of primordial follicles in the untreated controls and an increased number of primary follicles in the TubA group were observed 14 days after transplantation (Figure 3B-C). HE staining showed that the follicles continued to develop. There was an increase in the number of follicular granulosa cell layers in the TubA-treated group 14 days after transplantation (Figure 3D). We counted granulosa cell thickness in the TubA group and found a significant increase in follicles with oocyte diameters of 35-40, 40-45, 45-50, 50-55, and 55-60 μm (Figure 3E). These results indicated that TubA treatment could promote primordial follicle activation and follicle development *in vitro*.

The disruption of HIPPO signaling and short-duration AKT activator treatment (24 hours) can be used for *in vitro* activation of primordial follicles in clinical procedures to help patients with premature ovarian failure to bear children 28. In this study, we observed that decreasing HDAC6 during primordial follicle to primary follicle promoted primordial follicle activation (Figure S1G, Figure 3A-E), consistent with our previous report 52. Therefore, we conducted *in vitro* activation experiments of primordial follicles with shorter drug treatment times. We added TubA to the newborn mouse ovaries culture medium for 30 min, then transferred to the regular medium for 12 h or transplanted to immunodeficient mice under the kidney dorsal membrane for 14 days before collecting samples (Figure 3F). Ovary samples cultured for 12 h were collected for Western blotting to examine key molecules of the PI3K and mTOR signaling pathways. The results showed a significant increase in mTOR and p-mTOR, no change in AKT but a significant increase in p-AKT in the TubA group (Figure 3G). Immunofluorescence results revealed a substantial increase in cytoplasmic FOXO3a (CL-FOXO3a) in the TubA group (Figure 3H-I). These results suggested that a 30-minute TubA treatment could promote the activation of mouse primordial follicles *in vitro*.

Ovaries were collected 14 days after kidney dorsal membrane transplantation to observe the changes in ovarian volume and follicular structure. As shown in Figures 3J-K, the ovarian volume was significantly large in the TubA-treated group. hematoxylin stain of ovarian sections showed clusters of primordial follicles in the control group, which were not present in the TubA-treated group of activated follicles (Figure 3L). We also collected human ovarian cortical sections for similar experiments. The results showed that a 30-minute treatment with TubA also promoted human primordial follicle activation *in vitro* (Figure 3M). These data suggested that HDAC6 could promote primordial follicle activation and follicular development, and TubA, an HDAC6 inhibitor, could be used to target human primordial follicle activation *in vitro*.

### HDAC6 maintains primordial follicle dormancy and follicular reserve via the neuroligand/receptor signaling pathway

**Figure 4:**
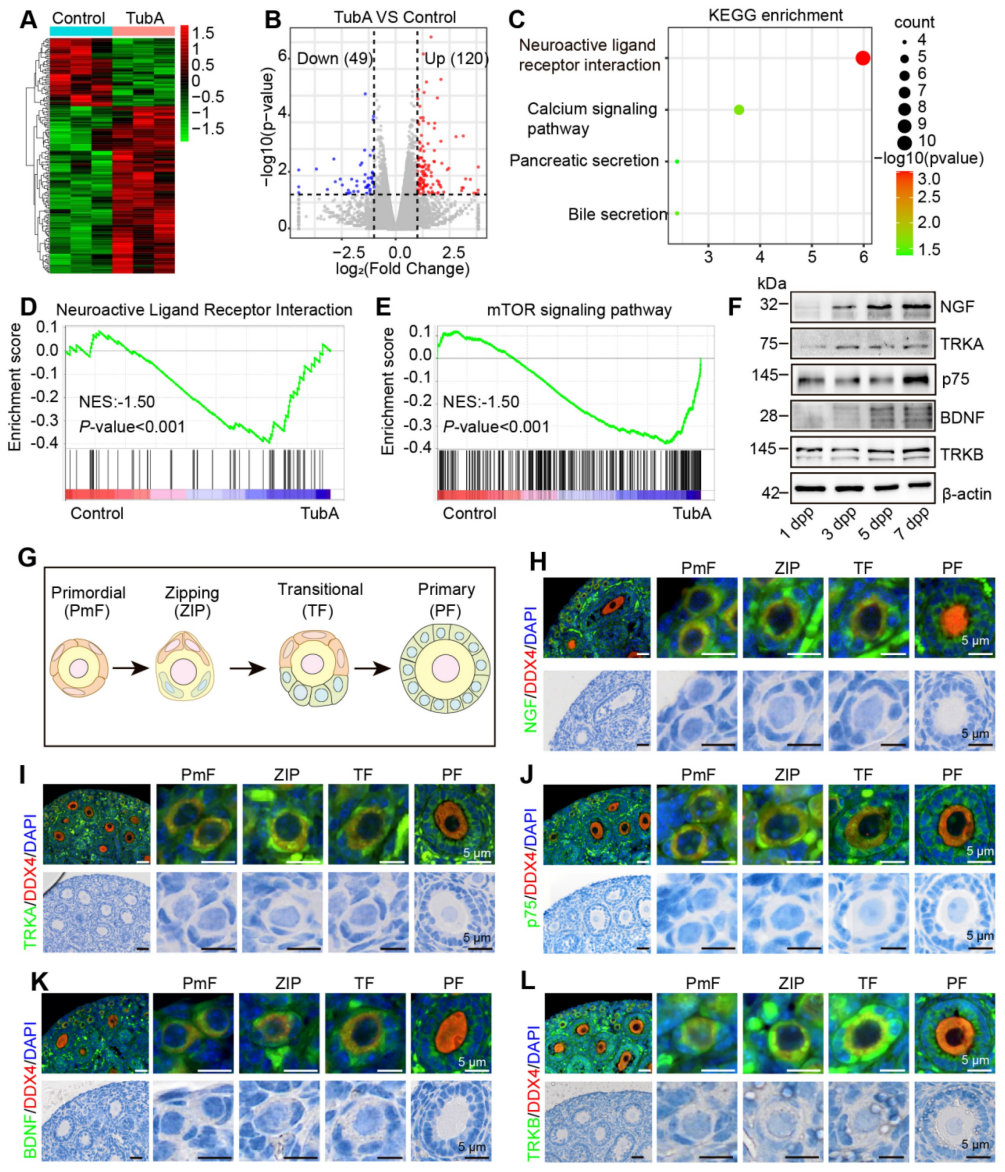
** HDAC6 regulates the expression of molecules related to the neuroactive ligand-receptor signaling pathway. (A)** Heat map of differentially expressed genes in the TubA group compared with the control group. **(B)** Volcano plot of differentially expressed genes in the TubA group compared with the control group. **(C)** KEGG enrichment of differentially expressed genes in the TubA group compared with the control group. **(D-E)** GSEA analysis of gene expression data. **(F)** Western blot of NGF, TRKA, p75, BDNF, and TRKB in newborn mouse ovaries at different time points. β-actin was used as an internal control. **(G)** Schematic diagram of PmF, ZIP, TF, and PF. **(H-L, upper)** BDNF, TRKB, NGF, TRKA, and p75 were examined by immunofluorescence in 7 dpp mouse ovarian sections. Signals were co-stained with DDX4 (red), the marker of oocytes. Nuclei were stained with DAPI (blue). Scale bars, 25 μm. **(H-L, lower)** HE staining of 7 dpp mouse ovarian sections.

We collected mouse ovaries cultured with TubA for transcriptome sequencing to investigate the mechanisms of HDAC6 regulation for maintaining primordial follicular dormancy and follicular reserve. The expression of 49 genes was down-regulated, and 120 genes were up-regulated in the TubA group compared to the control group (Figure 4A-B). KEGG enrichment analysis of the differentially expressed genes showed that they were mainly enriched in the signaling pathways of "neuroligand/receptor interactions," "calcium signaling," "paracrine," and "bile secretion" (Figure 4C). GSEA enrichment analysis showed significant enrichment of the "neuroligand/receptor signaling pathway" and "mTOR signaling pathway" (Figure 4D-E). Several studies have shown that the neuroligand/receptor family of NGF and its high-affinity receptor TRKA and low-affinity receptor p75, and BDNF and its receptor TRKB play essential roles in oogenesis and follicular development 30, 36, 45, 69. Therefore, we first analyzed the NGF, TRKA, p75, BDNF, TRKB, and TRKC expression in human follicular granulosa cells and oocytes at different developmental stages using published single-cell transcriptome sequencing data (GSE107746) 54. The results showed that the expression of NGF, TRKA and p75 was relatively low in human ovaries. p75 was increased in oocytes from primordial to primary follicles (Figure S6A-F). The expression of BDNF was high in all stages of follicular granulosa cells and oocytes during follicular development (Figure S6G-J). TRKC expression was extremely low in granulosa cells; it was highly expressed in ovarian oocytes and increased rapidly from the primordial follicle to the primary follicle stage and decreased gradually from primary follicles to the preovulatory follicles (Figure S6K-L).

BDNF and TRKB are highly expressed in the human ovary and have been shown to play significant roles in follicular development. Therefore, we performed a correlation analysis between HDAC6 and the expression of BDNF and TRKB in human follicular granulosa cells and oocytes at different developmental stages. A high and low mutually exclusive expression of HDAC6 and BDNF was observed in the same cell (Figure S7A-T). We collected 1, 3, 5, and 7 dpp mouse ovaries and examined NGF, TRKA, p75, BDNF, and TRKB expression patterns during primordial follicle activation. NGF protein expression gradually increased during primordial follicle activation, TRKA and p75 were also increased; BDNF and TRKB expression was also found to increase gradually (Figure 4F, Figure S8A-D).

We collected 7 dpp mouse ovaries for immunofluorescence to examine changes in the subcellular localization and expression of NGF, TRKA, p75, BDNF, and TRKB in different follicular stages. Figure 4G shows that NGF was expressed in the cytoplasm of oocytes, granulosa cells, and interstitial cells, and its expression in pre-granulosa cells increased during the development of the PmF into the ZIP and TF. Subsequently, NGF expression decreased during the development of the transitional follicle into a primary follicle. A similar trend was observed for TRKA, p75, BDNF, and TRKB expression, with maximum expression in the pre-granulosa cells of the ZIP and TF (Figure 4H-L). We further performed immunofluorescence using adult mouse ovaries and observed abundant expression of NGF, TRKA, p75, BDNF, and TRKB in interstitial cells (Figure S9A-E), consistent with previous reports 30. suggesting their involvement in primordial follicle activation and follicular development.

**Figure 5:**
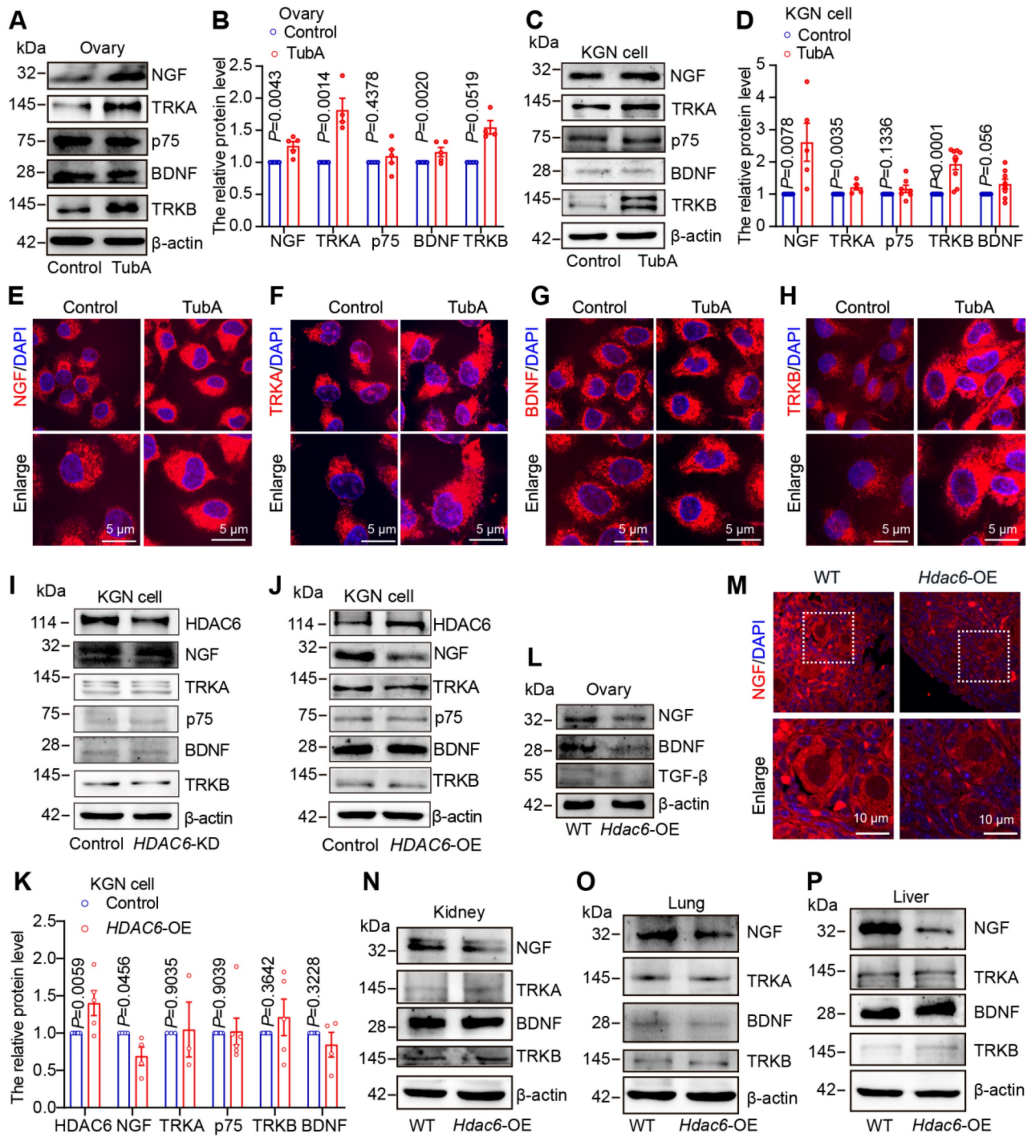
** HDAC6 suppresses protein levels of neuroreceptor ligand signaling pathway molecules. (A)** Western blot of NGF, TRKA, p75, BDNF, and TRKB in cultured mouse ovaries. The 3 dpp ovaries were cultured with or without TubA for 2 days. **(B)** Statistical analysis of (A). **(C)** Western blot of NGF, TRKA, p75, BDNF, and TRKB in TubA-treated KGN cells cultured with or without TubA for 24 h. **(D)** Statistical analysis of Figure C. **(E-H)** NGF, TRKA, TRKB, and BDNF were examined by immunofluorescence in TubA-treated KGN cells. KGN cells were cultured with or without TubA for 24 h. Nuclei were stained with DAPI (blue). Scale bars, 5 μm. **(I)** Western blot of NGF, TRKA, p75, BDNF, and TRKB in *HDAC6* knockdown (*HDAC6*-KD) KGN cells. **(J)** Western blot of NGF, TRKA, p75, BDNF, and TRKB in *HDAC6* overexpressing (*HDAC6*-OE) KGN cells. **(K)** Statistical analysis of Figure J. **(L)** Western blot of NGF, BDNF, and TGF-β in *Hdac6*-OE mouse ovaries. **(M)** NGF was examined by immunofluorescence in 7 dpp *Hdac6-OE* mouse ovarian sections. The NGF signal is labeled red. Nuclei were stained with DAPI (blue); scale bars, 10 μm. **(N-P)** Western blot of NGF, BDNF, TRKA, and TRKB in *Hdac6*-OE mouse kidney (N), lung (O), and liver (P).

Newborn mouse ovaries and KGN cells cultured with TubA were used to determine the changes in NGF, TRKA, p75, BDNF, and TRKB after HDAC6 inhibition. Western blotting showed that the protein expression levels of NGF, TRKA, BDNF, and TRKB were significantly elevated in the TubA-treated ovaries (Figure 5A-B) and KGN cells (Figure 5C-D); p75 expression was unchanged in ovarian cells. The NGF, TRKA, and TRKB expression showed a significant increase by immunofluorescence staining in TubA-treated KGN cells, while BDNF remained unchanged (Figure 5E-H, Figure S10A-D). Knockdown of *HDAC6* in KGN cells resulted in a decrease in HDAC6 protein expression, an increase in NGF expression, and unchanged protein expression levels of TRKA, p75, BDNF, and TRKB (Figure 5I). After overexpression of *HDAC6* in KGN cells, HDAC6 protein expression increased, while that of NGF decreased, and TRKA, p75, BDNF, and TRKB expression remained unchanged (Figure 5J-K).

Ovaries, kidneys, lungs, and livers from *Hdac6*-OE mice were collected and analyzed by Western blotting. The expression of NGF, BDNF and TGF-β was decreased in *Hdac6-OE* mouse ovaries (Figure 5L-M, Figure S10E-H). NGF expression was also reduced in the kidneys, lungs, and liver of *Hdac6*-OE mice, whereas TRKA, BDNF, and TRKB protein expression levels were unchanged (Figure 5N-P, Figure S10I). These results indicated that HDAC6 may maintain mouse primordial follicle dormancy by inhibiting the expression of NGF and BDNF. Furthermore, HDAC6 regulates NGF expression across multiple organs.

**Figure 6:**
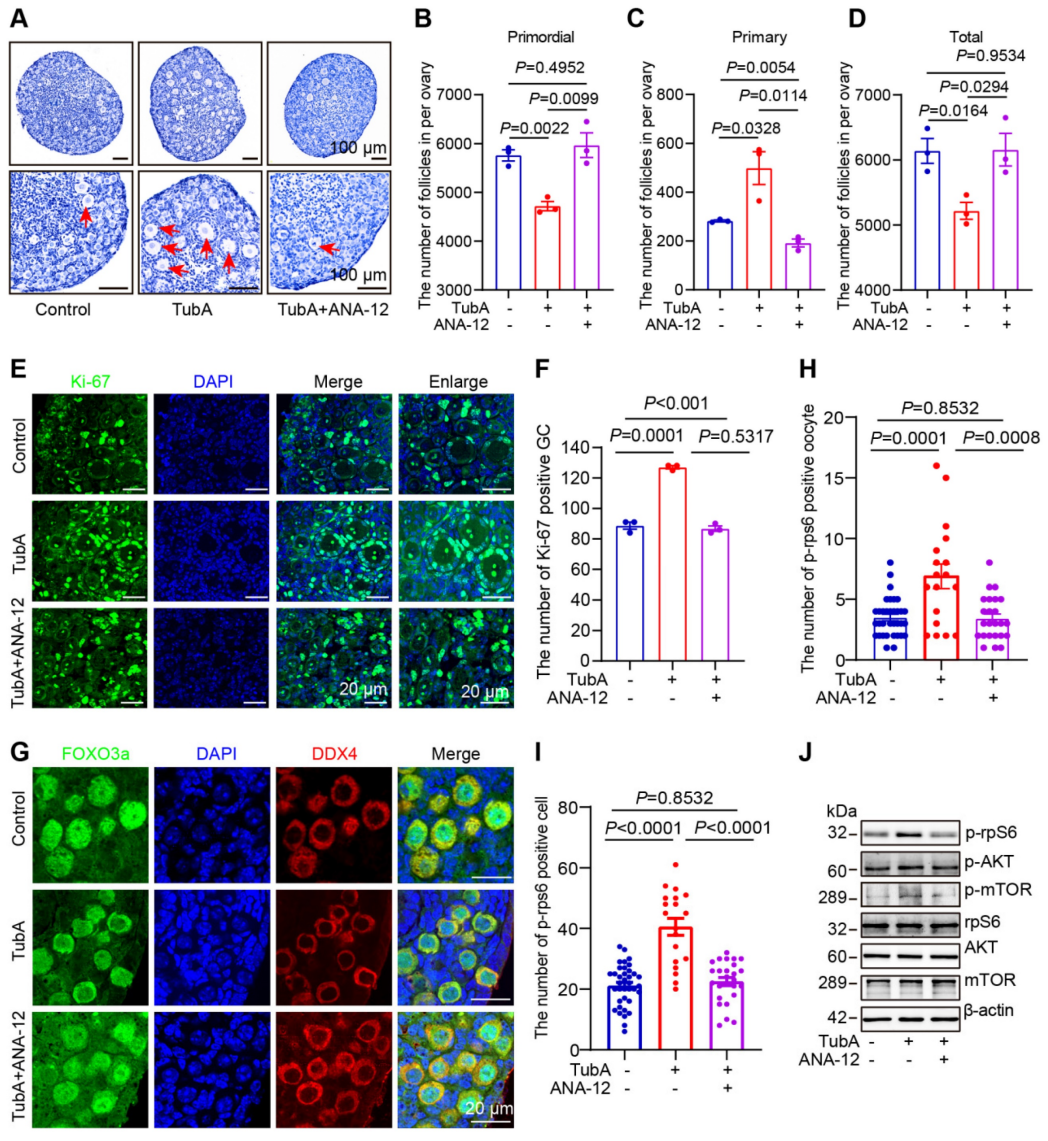
** HDAC6 regulates follicle reserve via the neuroreceptor ligand signaling pathway. (A)** Hematoxylin staining of ovarian sections from TubA and ANA-12 treated ovaries. The 3 dpp ovaries were cultured for 3 days. **(B-D)** Whole ovary follicle counts from primordial, primary, and total follicles. **(E)** The Ki-67 immunofluorescence (green) was examined in the TubA- and ANA-12-treated ovaries. The 3 dpp ovaries were cultured for 2 days; Nuclei were stained with DAPI (blue). **(F)** Statistical analysis of Ki-67-positive granulosa cells in TubA and ANA-12 treated ovaries. **(G)** FOXO3a immunofluorescence (red) was examined in TubA- and ANA-12-treated ovaries. DDX4 (red) is the marker for oocytes. Nuclei were stained with DAPI (blue). **(H-I)** Statistical analysis of p-rpS6-positive granulosa cells and oocytes in TubA and ANA-12 treated ovaries. The 3 dpp ovaries were cultured for 2 days. **(J)** Western blot of mTOR, p-mTOR, rpS6, p-rpS6, AKT, and p-AKT in TubA and ANA-12 treated ovaries. The 3 dpp ovaries were cultured for 2 days.

We cultured newborn mouse ovaries with TubA, the NGF receptor inhibitor K252a, and the BDNF receptor inhibitor ANA-12. HE staining followed by ovarian follicle counting showed that adding TubA to cultured newborn mouse ovaries promoted primordial follicle activation. The TubA effect was reversed when TubA and ANA-12 or TubA and K252a were added to cultured mouse ovaries (Figure 6A-D, Figure S11A-D). The immunofluorescence results showed that the number of Ki-67-positive granulosa cells was significantly increased in the TubA group relative to the control group. There was no significant difference in the number of Ki-67 positive granulosa cells between the TubA+ANA-12 and control groups (Figure 6E-F). CL-FOXO3a was increased in primordial follicular oocytes in the TubA group compared to the control group. There was no significant difference in CL-FOXO3a in primordial follicle oocytes in the TubA+ANA-12 and control groups (Figure 6G). More p-rpS6 positive oocytes and granulosa cells were observed in the TubA group compared to the control group. There was no significant difference in the number of p-rpS6 positive oocytes and granulosa cells in TubA+ANA-12 and the control groups (Figure 6H-I). Consistent with our previous study 52, sequencing results in Figure 4E, indicated regulation of the mTOR signaling pathway by HDAC6 during primordial follicle activation. Western blotting showed no change in mTOR, but p-mTOR and p-AKT were significantly increased in the TubA group compared to the control group. Furthermore, mTOR, p-mTOR, AKT, and p-AKT were not changed in the TubA+ANA-12 group compared to the control group (Figure 6J, Figure S11E-J). These results suggested that HDAC6 inhibits the mTOR signaling pathway via NGF/BDNF, thereby maintaining mouse primordial follicle dormancy.

### HDAC6 forms a protein complex with NGF, BDNF, TGF-β, and TSC2 to maintain mouse primordial follicle dormancy

**Figure 7:**
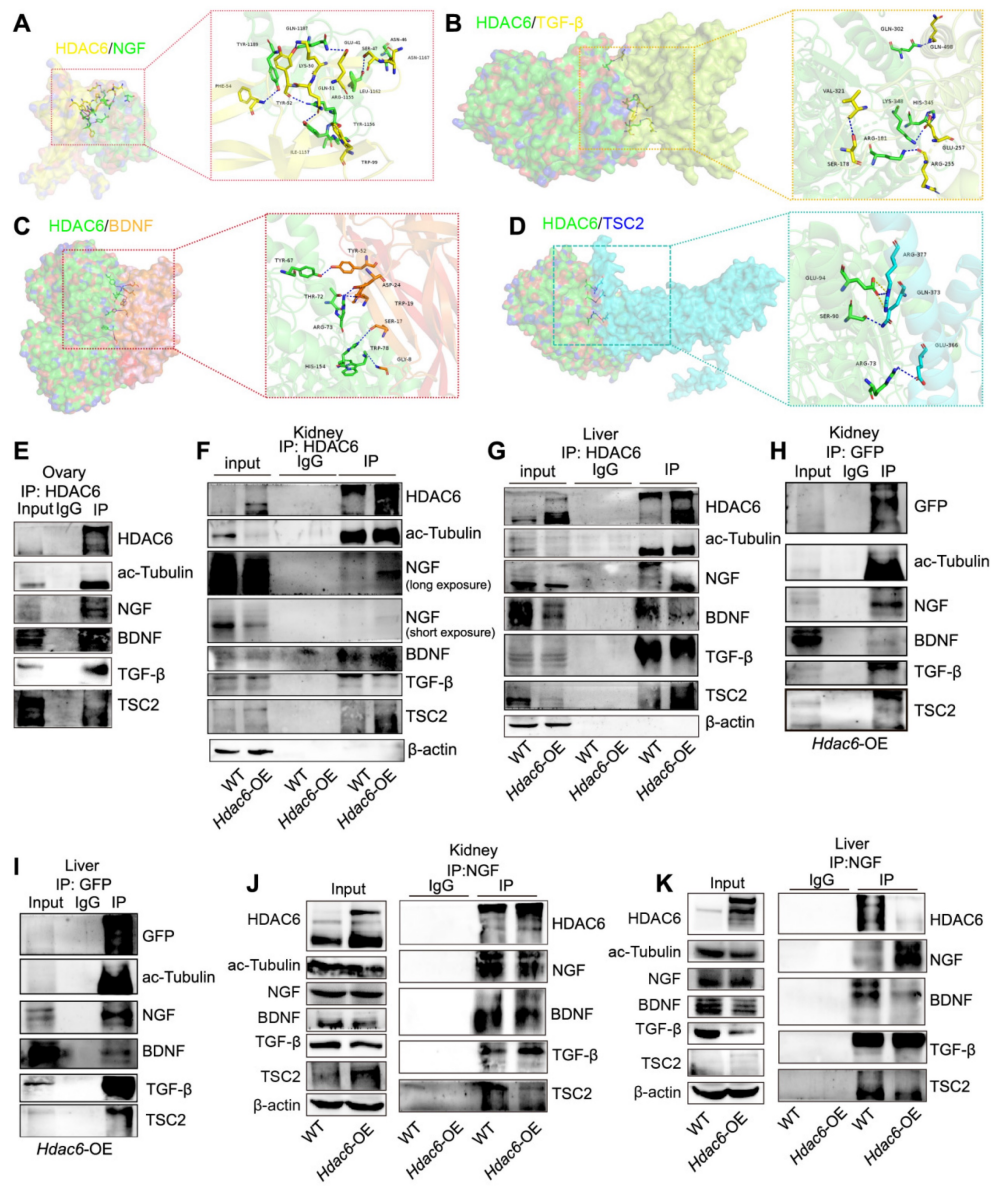
** HDAC6 interacts with NGF, BDNF, TGF-β, and TSC2.** Protein-protein molecular docking demonstrated the binding between **(A)** HDAC6 and NGF **(B)** HDAC6 and TGF-β **(C)** HDAC6 and BDNF **(D)** HDAC6 and TSC2. **(E)** Interactions between HDAC6 and NGF, BDNF, TGF-β, TSC2, and ac-Tubulin were detected in wild-type ovaries by Co-IP with NGF antibody. Approximately 10% of lysate was loaded as input. **(F-K)** Interactions between HDAC6 and NGF, BDNF, TGF-β, TSC2, and ac-Tubulin were detected in the wild-type and *Hdac6*-OE kidney and liver by Co-IP with **(F-G)** HDAC6 antibody, **(H-I)** GFP antibody, and **(J-K)** NGF antibody. Approximately 5% of lysate was loaded as input.

HDAC6, TGF-β, NGF, and BDNF are known to regulate primordial follicular reserve and primordial follicle activation through the mTOR signaling pathway 30, 31, 52. TSC1/2 acts as a negative regulator of mTORC1, and TSC1/2 deletion or mutations lead to hyperactivation of primordial follicles 70, 71. When HDAC6 was inhibited or knocked down, TGF-β, NGF, and BDNF expression decreased, and TSC2 expression increased (Figure S12A-B). HDAC6, TGF-β, NGF, BDNF, and TSC2 are located in the cytoplasm. We speculated that there are protein interactions between HDAC6 and TSC2, TGF-β, NGF, and BDNF during primordial follicle activation. Protein molecular docking analysis showed that HDAC6 interacted with TSC2, TGF-β, NGF, and BDNF (Figure 7A-D). Since immunofluorescence results showed co-localization of TGF-β and NGF (Figure S12C-D), we performed Co-IP analysis of HDAC6 and NGF in ovarian tissues and KGN cells, respectively. The Co-IP analysis results showed that direct protein interactions were detected between HDAC6 and TGF-β, NGF, and BDNF. HDAC6 and ac-Tubulin served as positive controls in these experiments (Figure 7E, Figure S12E-G). Commercial antibodies to HDAC6, NGF, and BDNF are less bioavailable, and the neonatal mouse ovary tissue is small. It was challenging to perform Co-IP analysis in small neonatal mouse ovaries using commercial antibodies with limited bioavailability. Since HDAC6 and NGF expression was high in the kidney and liver (Figure S12H-K), and HDAC6 regulation of the expression of these molecules was conserved across different organs (Figure 5N-P), Co-IP analysis was carried out using kidney and liver tissues. The results showed that direct protein interactions between endogenous HDAC6 and TSC2, TGF-β, NGF, and BDNF (Figure 7F-G). Furthermore, we established HDAC6 transgenic mice fused with a GFP tag (Figure 2A, Figure S3A-G), and collected kidneys and livers from *Hdac6*-OE mice for Co-IP of GFP. The results demonstrated that exogenous HDAC6 directly interacted with TGF-β, NGF, and BDNF (Figure 7H-I). Exogenous NGF could also directly interact with HDAC6, TGF-β, BDNF, and TSC2 (Figure 7J-K). These results indicated that HDAC6 forms protein complexes with NGF, BDNF, TGF-β, and TSC2 to maintain mouse primordial follicle dormancy.

### Deacetylation of NGF by HDAC6 facilitates its ubiquitination and decreases NGF protein stability

**Figure 8:**
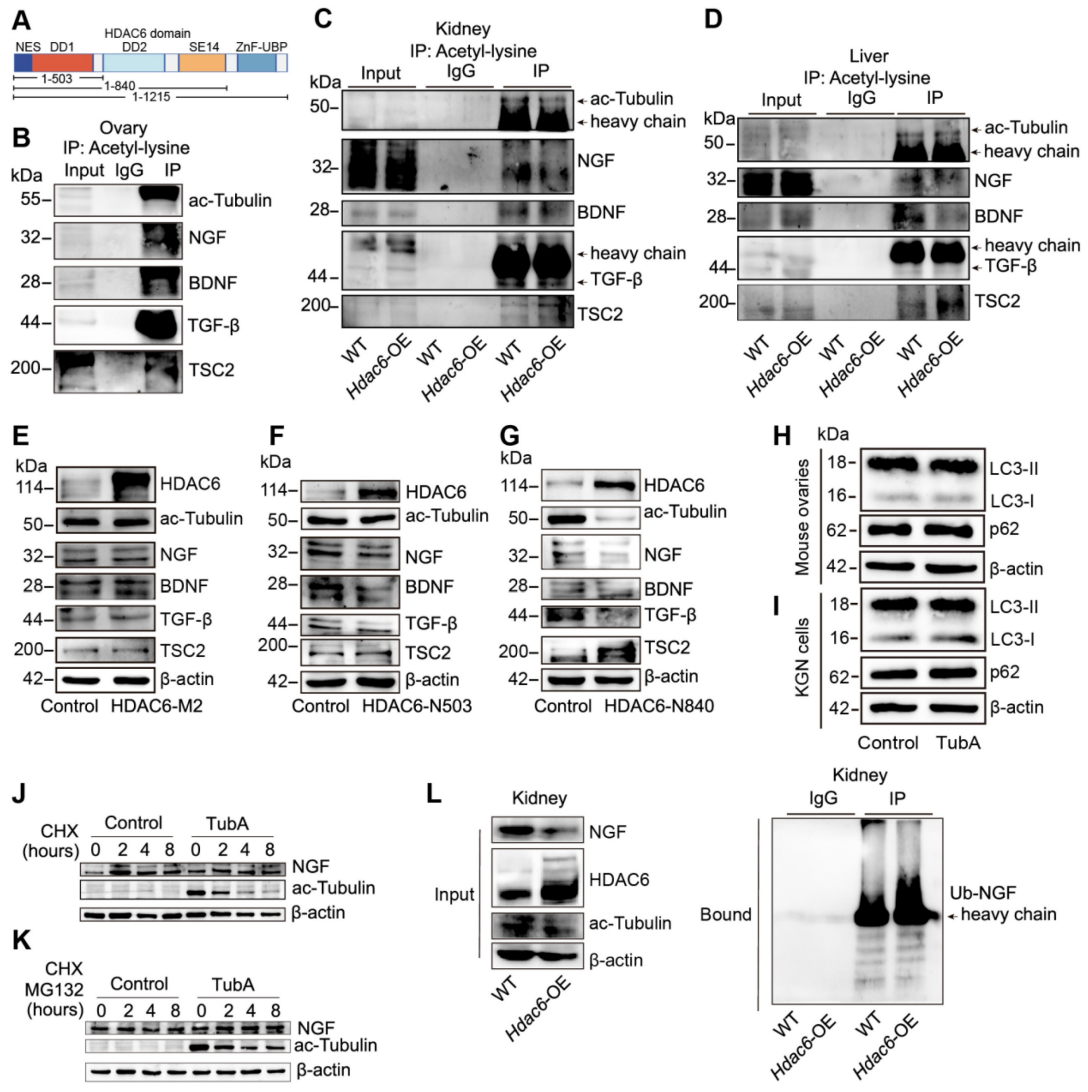
** Deacetylation of NGF by HDAC6 facilitates ubiquitination and decreases its stability. (A)** Schematic diagram of HDAC6 protein and three HDAC6 deletion mutants. DD1: first deacetylase domain. DD2: second deacetylase domain. SE14: Ser/Glu-containing tetradecapeptide repeats domain. ZnF-UBP: ubiquitin-binding zinc finger domain. Acetylation modification of ac-Tubulin, NGF, BDNF, TGF-β, and TSC2 was detected in **(B)** wild-type ovaries and **(C-D)** wild-type and *Hdac6*-OE kidney and liver by Co-IP of acetyl-lysine antibody **(E-G)** Western blot of HDAC6, ac-Tubulin, NGF, BDNF, TGF-β, and TSC2 in HDAC6-M2, HDAC6-N503, and HDAC6-N840 overexpressing cells. HDAC6-M2: deacetylation catalytic activity defective mutant of HDAC6 (H216/611A), HDAC6-N503: DD1 domain deletion mutant of HDAC6 from 1-503, HDAC6-N840: DD1 and DD2 domain deletion mutant of HDAC6 from 1-840 **(H-I)** Western blot of p62 and LC3B in TubA-treated ovaries and KGN cells. 3 dpp ovaries were cultured with TubA for 2 days. KGN cells were cultured with TubA for 24 h. **(J)** Western blot of NGF and ac-Tubulin in TubA-treated KGN cells. TubA group cells were cultured with TubA for 24 h. Control and TubA group cells were treated with 20 μM CHX for indicated times. **(K)** Western blot of NGF and ac-Tubulin in TubA-treated-KGN cells. TubA group cells were cultured with TubA for 24 h. Subsequently, the control group and TubA group cells were treated with 20 μM CHX and 25 μM MG132 for indicated times. **(L)** Ubiquitination modification of NGF was detected in the wild-type and *Hdac6*-OE kidney by Co-IP with ubiquitination antibody.

HDAC6 has two deacetylated and one ubiquitinated structural domain (Figure 8A) 50. We used acetyl-lysine antibody for Co-IP analysis in ovarian tissues. Figure 8B shows the acetylation modification of TSC2, NGF, BDNF, and TGF-β. We further investigated whether HDAC6 could regulate the acetylation ofTSC2, NGF, BDNF, and TGF-β. Since obtaining sufficient small-sized ovarian tissues from *Hdac6*-OE mice is challenging, we collected kidneys and livers from wild-type and *Hdac6*-OE mice for Co-IP with acetyl-lysine antibody. The results showed that the acetylation levels of TSC2, NGF, BDNF, and TGF-β were reduced in kidney and liver tissues of *Hdac6*-OE mice (Figure 8C-D). These results suggested direct regulation of TSC2, NGF, BDNF, and TGF-β acetylation modification by HDAC6.

We constructed HDAC6-M2 peptide by mutating the deacetylation structural domains 503 and 840 of HDAC6 50 and found that HDAC6 expression was significantly increased while ac-Tubulin expression was unchanged in the HDAC6-M2 group. Interestingly, the expression of TSC2, NGF, BDNF, and TGF-β did not change in the HDAC6-M2 group (Figure 8E). When the N-terminal HDAC6 mutant (1-503) containing the first deacetylase domain (DD1) was overexpressed in HEK293T cells, the protein level of HDAC6 was increased, ac-Tubulin was unchanged, and TSC2, NGF, BDNF, and TGF-β were decreased (Figure 8F). When the N-terminal HDAC6 mutant (1-840), containing, first deacetylase domain, the second deacetylase domains (DD2), was overexpressed in HEK293T cells, the HDAC6 protein level was increased and ac-Tubulin, TSC2, NGF, BDNF, and TGF-β levels were decreased (Figure 8G). These results suggested that HDAC6-dependent deacetylation activity regulated the expression of TSC2, NGF, BDNF, and TGF-β, which were degraded following deacetylation by HDAC6.

HDAC6 degrades proteins via autophagy or the ubiquitinated proteasome pathway 51, 72. Since lysine acetylation is vastly involved in post-translational modifications, such as ubiquitination 73, we investigated whether HDAC6 regulated TSC2, NGF, BDNF, and TGF-β transcriptional levels, autophagy, or the acetylation and ubiquitination crosstalk pathway. qPCR results showed that *TSC2*, *NGF*, *BDNF*, and T*GF-β* mRNA levels did not change in the TubA-treated ovaries and KGN cells (Figure S12L-M). The protein levels of p62 and LC3B were unchanged in the TubA-treated ovaries and KGN cells (Figure 8H-I, Figure S12N-O).

Next, we utilized CHX to inhibit protein synthesis in KGN cells. The Western blot results indicates that NGF decreased in the control group cells but remained stable in TubA-treated cells (Figure 8J). Further, we used MG132 to inhibit 26S proteasome and found restitution of NGF protein expression (Figure 8K). These data suggested that HDAC6 induced a decrease in NGF by the ubiquitination-proteasomal degradation pathway. Consistent with this observation in KGN cells, the *Hdac6*-OE kidney contained more multi-ubiquitinated NGF than control cells (Figure 8L). Therefore, the acetylation and ubiquitination crosstalk by HDAC6 may be involved in NGF protein stability regulation.

It is plausible that the physiological significance of the high expression of HDAC6 in the vast majority of primordial follicles is to reduce NGF expression and prevent premature primordial follicle activation. When HDAC6 was decreased in primordial follicles, NGF expression was increased, promoting primordial follicle activation. Our studies and those of others have found that primordial follicle activation and follicular development are faster in C3H mice than in C57 mice at the same time points (Figure S13A-B) 74. In the present study, we found that HDAC6 expression was decreased in TF follicles from C57 mouse ovaries. Interestingly, HDAC6 was already decreased in ZIP in C3H mouse ovaries (Figure S13C-D). Meanwhile, NGF expression was increased during the transition from primordial to activated follicles and was higher in C3H mice than in C57 mouse ovaries, especially in ZIP and TF follicles (Figure S13E-F). These results suggested that reduced HDAC6 expression in dormant primordial follicles upregulates NGF expression and promotes primordial follicle activation.

**Figure 9:**
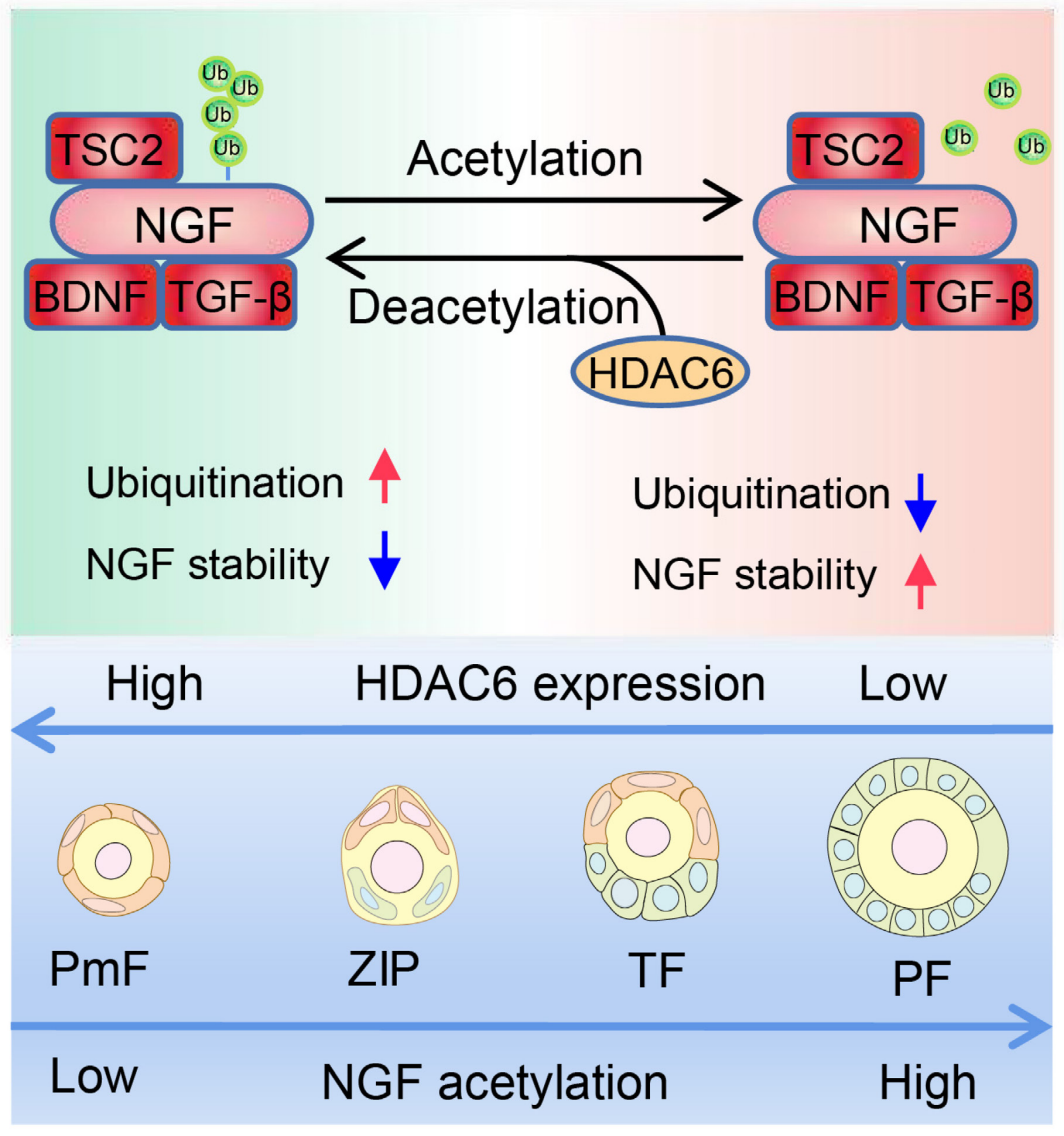
** Working Model: HDAC6 maintains primordial follicle dormancy.** HDAC6 is highly expressed in primordial follicles. HDAC6 forms a protein complex with NGF, BDNF, TGF-β, and TSC2. HADC6 deacetylates NGF, and acetylated NGF is degraded by ubiquitination. Low levels of NGF cannot activate the mTOR signaling pathway, rendering primordial follicles dormant. HDAC6 gradually decreases during the development of primordial to primary follicles. NGF is not modified by deacetylation and cannot be degraded by ubiquitination. The elevated NGF activates the mTOR signaling pathway; thus, primordial follicles are activated to develop into primary follicles.

In conclusion, HDAC6 is a key factor in maintaining female fertility. High expression of HDAC6 in dormant primordial follicles deacetylates NGF and promotes its ubiquitination and degradation, keeping NGF at a low level in dormant primordial follicles, sustaining their dormancy and, and maintaining extended fertility (Figure 9).

## Discussion

The size of the primordial follicle pool and its activation rate are critical determinants of female fertility length. In mammals, only a limited number of primordial follicles are selectively activated, while most remain dormant 10, 11. In this study, we have shown that overexpression of *Hdac6* enlarged the primordial follicle pool, reduced follicle depletion, and prolonged fertility in female mice. We characterized the role of HDAC6 in primordial follicle activation and identified NGF, BDNF, TGF-β, and TSC2 as new interactors and substrates. Mechanistic studies showed that HDAC6-mediated deacetylation of NGF instigated its degradation by the ubiquitin-proteasome system. Thus, high expression of HDAC6 decreased NGF expression in primordial follicles, sustaining their dormancy and thus maintaining female fertility.

HDAC6 expression decreases with reproductive aging in the naturally aging mouse model, cisplatin-induced premature ovarian failure (POF) model, human ovarian granulosa cell model with diminished ovarian reserve (POF), and cisplatin-treated human ovarian granulosa cell model. Analysis of previously published sequencing data from human and mouse ovaries revealed that HDAC6 expression was relatively high in primordial follicles 52, 54, 66. It has also been shown that overexpression of *Hdac6* prolongs mouse fertility 53. These results suggest that HDAC6 plays a vital role in fertility maintenance. The biological significance of high expression of HDAC6 in primordial follicles is to maintain primordial follicle quiescence, thereby preserving ovarian reproductive life.

Although there are many members of the neurotrophic factor family, the total and polysomal NGF RNA is predominantly upregulated in interstitial cells after ovarian injury to promote selective activation of primordial follicles around the injury. BDNF is also upregulated in injured adult ovaries 30. TRKA, as an NGF ligand, promotes ovarian ovulation and angiogenesis. TRKB is a ligand for BDNF, and TRKB knockout mice have been shown to lose many follicles by 4 dpp 36, 39, 41. TRKB agonistic antibody promotes follicular development and improves fertility in mouse models of natural aging and chemotherapy-induced premature ovarian failure 45. These studies illustrated that NGF/TRKA and BDNF/TRKB are indispensable for follicular development and reserve.

Our results demonstrated that HDAC6 forms a complex with NGF and BDNF to regulate primordial follicle activation. Short-term inhibition of HDAC6 promotes the activation of mouse and human primordial follicles in vitro, and the activated follicles can develop normally. Our study suggests that HDAC6, NGF/TRKA, and BDNF/TRKB can be used as targets to treat follicular development disorders, decreased follicular reserve, and other related diseases.

We have previously shown that mTORC1, especially in pre-granulosa cells, is indispensable for primordial follicle activation in mouse ovaries 20, 52. HDAC6, TGF-β, and NGF maintain primordial follicle reserve and promote primordial follicle selective activation through upregulation of the mTOR signaling pathway 30, 31. In the present study, we have expanded upon this concept and shown that HDAC6 forms a protein complex with TSC2, TGF-β, NGF, and BDNF to control primordial follicle dormancy and activation state transition. Growth factors, including TGF-β, NGF, and BDNF, promote primordial follicle activation. Furthermore, high levels of HDAC6 in primordial follicles maintain the expression of TGF-β, NGF, and BDNF at relatively low levels. Reduced HDAC6 expression in primordial follicles abolishes deacetylation modification of TGF-β, NGF, and BDNF and ubiquitination degradation of these growth factors, increasing their expression and thus promoting primordial follicle activation. The mTORC1 pathway is responsive to environmental factors, such as nutrients, oxygen, energy, and growth factors, and regulates cell growth and metabolism 20. Each follicle is in a relatively independent microenvironment, and changes in the follicular microenvironment may directly affect follicular developmental fate. As an epitope modification molecule, whether HDAC6 can sense the changes of TGF-β, NGF, and BDNF in the primordial follicle microenvironment and thus regulate the growth and metabolism of primordial follicles is a direction we need to focus on in the future.

Two waves of primordial follicles are present in the ovary, and there is heterogeneity in the activation of primordial follicles: The first wave of primordial follicle activation is independent of pre-granulosa cells and is initiated by oocytes 74. The activation of the second wave of primordial follicles is strictly initiated by the pre-granulosa cells 20. In the present study, we used a transgenic mouse model overexpressing *Hdac6* throughout the body. It is unclear exactly how HDAC6 regulates follicular development and whether overexpression of *Hdac6* in other organs affects ovarian development. Also, there are no studies showing changes in follicular development following HDAC6 deletion. Conditional knockout or overexpression mouse models of *Hdac6* are essential for an in-depth understanding of its role in follicular development.

## Supplementary Material

Supplementary figures and tables.

## References

[1] Monget P, McNatty K, Monniaux D (2021). The Crazy Ovary. Genes (Basel).

[2] Matzuk MM, Lamb DJ (2008). The biology of infertility: research advances and clinical challenges. Nat Med.

[3] Inhorn MC, Patrizio P (2015). Infertility around the globe: new thinking on gender, reproductive technologies and global movements in the 21st century. Hum. Reprod. Update.

[4] Carson SA, Kallen AN (2021). Diagnosis and management of infertility: a review. JAMA.

[5] Dougherty MP, Poch AM, Chorich LP, Hawkins ZA, Xu H, Roman RA (2023). Unexplained female infertility associated with genetic disease variants. N. Engl. J. Med.

[6] Ennab F, Atiomo W (2023). Obesity and female infertility. Best Pract Res Clin Obstet Gynaecol.

[7] He M, Zhang T, Yang Y, Wang C (2021). Mechanisms of oocyte maturation and related epigenetic regulation. Front Cell Dev Biol.

[8] Moghadam ARE, Moghadam MT, Hemadi M, Saki G (2022). Oocyte quality and aging. JBRA Assist Reprod.

[9] Henry L, Labied S, Jouan C, Nisolle M (2022). Preservation of female fertility: The current therapeutic strategy. Int J Gynaecol Obstet.

[10] Zhang H, Liu K (2015). Cellular and molecular regulation of the activation of mammalian primordial follicles: somatic cells initiate follicle activation in adulthood. Hum Reprod Update.

[11] Zhang T, He M, Zhang J, Tong Y, Chen T, Wang C (2023). Mechanisms of primordial follicle activation and new pregnancy opportunity for premature ovarian failure patients. Front Physiol.

[12] Zhang H, Panula S, Petropoulos S, Edsgärd D, Busayavalasa K, Liu L (2015). Adult human and mouse ovaries lack DDX4-expressing functional oogonial stem cells. Nat Med.

[13] Reddy P, Liu L, Adhikari D, Jagarlamudi K, Rajareddy S, Shen Y (2008). Oocyte-specific deletion of Pten causes premature activation of the primordial follicle pool. Science.

[14] Castrillon DH, Miao L, Kollipara R, Horner JW, DePinho RA (2003). Suppression of ovarian follicle activation in mice by the transcription factor Foxo3a. Science.

[15] Ren Y, Suzuki H, Jagarlamudi K, Golnoski K, McGuire M, Lopes R (2015). Lhx8 regulates primordial follicle activation and postnatal folliculogenesis. BMC Biol.

[16] Wang Z, Liu C-Y, Zhao Y, Dean J (2020). FIGLA, LHX8 and SOHLH1 transcription factor networks regulate mouse oocyte growth and differentiation. Nucleic Acids Res.

[17] Saatcioglu HD, Cuevas I, Castrillon DH (2016). Control of oocyte reawakening by Kit. PLoS Genet.

[18] Yan H, Zhang J, Wen J, Wang Y, Niu W, Teng Z (2018). CDC42 controls the activation of primordial follicles by regulating PI3K signaling in mouse oocytes. BMC Biol.

[19] Yan H, Wen J, Zhang T, Zheng W, He M, Huang K (2019). Oocyte-derived E-cadherin acts as a multiple functional factor maintaining the primordial follicle pool in mice. Cell Death Dis.

[20] Zhang H, Risal S, Gorre N, Busayavalasa K, Li X, Shen Y (2014). Somatic cells initiate primordial follicle activation and govern the development of dormant oocytes in mice. Current Biol.

[21] De Baere E, Lemercier B, Christin-Maitre S, Durval D, Messiaen L, Fellous M (2002). FOXL2 mutation screening in a large panel of POF patients and XX males. J Med Genet.

[22] Granados-Aparici S, Hardy K, Franks S, Sharum IB, Waite SL, Fenwick MA (2019). SMAD3 directly regulates cell cycle genes to maintain arrest in granulosa cells of mouse primordial follicles. Sci Rep.

[23] Habara O, Logan CY, Kanai-Azuma M, Nusse R, Takase HM (2021). WNT signaling in pre-granulosa cells is required for ovarian folliculogenesis and female fertility. Development.

[24] Schmidt D, Ovitt CE, Anlag K, Fehsenfeld S, Gredsted L, Treier A-C (2004). The murine winged-helix transcription factor Foxl2 is required for granulosa cell differentiation and ovary maintenance. Development.

[25] Zhang T, Du X, Zhao L, He M, Lin L, Guo C (2019). SIRT1 facilitates primordial follicle recruitment independent of deacetylase activity through directly modulating Akt1 and mTOR transcription. FASEB J.

[26] Zhang T, Lin H, Ren T, He M, Zheng W, Tong Y (2024). ROCK1 is a multifunctional factor maintaining the primordial follicle reserve and follicular development in mice. Am J Physiol Cell Physiol.

[27] Zheng W, Zhang T, Zhao T, Zhu Z, Qin S, Yan H (2023). cAMP controls the balance between dormancy and activation of primordial follicles in mouse ovaries. PNAS Nexus.

[28] Kawamura K, Cheng Y, Suzuki N, Deguchi M, Sato Y, Takae S (2013). Hippo signaling disruption and Akt stimulation of ovarian follicles for infertility treatment. Proc Natl Acad Sci U S A.

[29] Rajareddy S, Reddy P, Du C, Liu L, Jagarlamudi K, Tang W (2007). p27kip1 (cyclin-dependent kinase inhibitor 1B) controls ovarian development by suppressing follicle endowment and activation and promoting follicle atresia in mice. Mol Endocrinol.

[30] He Y, Peng X, Wu T, Yang W, Liu W, Zhang J (2017). Restricting the induction of NGF in ovarian stroma engenders selective follicular activation through the mTOR signaling pathway. Cell Death Dis.

[31] Wang Z-P, Mu X-Y, Guo M, Wang Y-J, Teng Z, Mao G-P (2014). Transforming growth factor-β signaling participates in the maintenance of the primordial follicle pool in the mouse ovary. J Biol Chem.

[32] Zhang X, Zhang W, Wang Z, Zheng N, Yuan F, Li B (2022). Enhanced glycolysis in granulosa cells promotes the activation of primordial follicles through mTOR signaling. Cell Death Dis.

[33] Knight PG, Glister C (2006). TGF-β superfamily members and ovarian follicle development. Reprod.

[34] Hardy K, Mora JM, Dunlop C, Carzaniga R, Franks S, Fenwick MA (2018). Nuclear exclusion of SMAD2/3 in granulosa cells is associated with primordial follicle activation in the mouse ovary. J Cell Sci.

[35] Ojeda SR, Romero C, Tapia V, Dissen GA (2000). Neurotrophic and cell-cell dependent control of early follicular development. Mol Cell Endocrinol.

[36] Kerr B, Garcia-Rudaz C, Dorfman M, Paredes A, Ojeda SR (2009). NTRK1 and NTRK2 receptors facilitate follicle assembly and early follicular development in the mouse ovary. Reprod.

[37] Dorfman MD, Garcia-Rudaz C, Alderman Z, Kerr B, Lomniczi A, Dissen GA (2014). Loss of Ntrk2/Kiss1r signaling in oocytes causes premature ovarian failure. Endocrinol.

[38] Alves AMCV, Chaves RN, Figueiredo JR, Lima LF, Matos HMT, Rodrigues APR (2013). Role of nerve growth factor (NGF) and its receptors in folliculogenesis. Zygote.

[39] Dorfman MD, Kerr B, Garcia-Rudaz C, Paredes AH, Dissen GA, Ojeda SR (2011). Neurotrophins acting via TRKB receptors activate the JAGGED1-NOTCH2 cell-cell communication pathway to facilitate early ovarian development. Endocrinol.

[40] Salas C, Julio-Pieper M, Valladares M, Pommer R, Vega M, Mastronardi C (2006). Nerve growth factor-dependent activation of trkA receptors in the human ovary results in synthesis of follicle-stimulating hormone receptors and estrogen secretion. J Clin Endocrinol Metab.

[41] Dissen GA, Romero C, Hirshfield AN, Ojeda SR (2001). Nerve growth factor is required for early follicular development in the mammalian ovary. Endocrinol.

[42] Ren L, Medan MS, Weng Q, Jin W, Li C, Watanabe G (2005). Immunolocalization of nerve growth factor (NGF) and its receptors (TrkA and p75LNGFR) in the reproductive organs of shiba goats. J Reprod Dev.

[43] Levanti MB, Germanà A, Abbate F, Montalbano G, Vega JA, Germanà G (2005). Brief communication: TrkA and p75NTR in the ovary of adult cow and pig. J Anat.

[44] Zheng X, Chen L, Chen T, Cao M, Zhang B, Yuan C (2023). The Mechanisms of BDNF promoting the proliferation of porcine follicular granulosa cells: role of miR-127 and involvement of the MAPK-ERK1/2 pathway. Anim.

[45] Qin X, Zhao Y, Zhang T, Yin C, Qiao J, Guo W (2022). TrkB agonist antibody ameliorates fertility deficits in aged and cyclophosphamide-induced premature ovarian failure model mice. Nat Commun.

[46] Kawaguchi Y, Kovacs JJ, McLaurin A, Vance JM, Ito A, Yao T-P (2003). The deacetylase HDAC6 regulates aggresome formation and cell viability in response to misfolded protein stress. Cell.

[47] Osseni A, Ravel-Chapuis A, Thomas J-L, Gache V, Schaeffer L, Jasmin BJ (2020). HDAC6 regulates microtubule stability and clustering of AChRs at neuromuscular junctions. J of Cell Biol.

[48] Hubbert C, Guardiola A, Shao R, Kawaguchi Y, Ito A, Nixon A (2002). HDAC6 is a microtubule-associated deacetylase. Nature.

[49] Kovacs JJ, Murphy PJM, Gaillard S, Zhao X, Wu J-T, Nicchitta CV (2005). HDAC6 regulates Hsp90 acetylation and chaperone-dependent activation of glucocorticoid receptor. Mol Cell.

[50] Deng Y, Gao J, Xu G, Yao Y, Sun Y, Shi Y (2022). HDAC6-dependent deacetylation of AKAP12 dictates its ubiquitination and promotes colon cancer metastasis. Cancer Lett.

[51] Lee JY, Koga H, Kawaguchi Y, Tang W, Wong E, Gao YS (2010). HDAC6 controls autophagosome maturation essential for ubiquitin-selective quality-control autophagy. EMBO J.

[52] Zhang T, He M, Zhao L, Qin S, Zhu Z, Du X (2021). HDAC6 regulates primordial follicle activation through mTOR signaling pathway. Cell Death Dis.

[53] Zhang X, Yang J, Wang H, Guo R, Yin Y, Zhang D (2017). Overexpression of Hdac6 extends reproductive lifespan in mice. Protein Cell.

[54] Zhang Y, Yan Z, Qin Q, Nisenblat V, Chang H-M, Yu Y (2018). Transcriptome landscape of human folliculogenesis reveals oocyte and granulosa cell interactions. Mol Cell.

[55] Zhang X, Liu G, Zhang N, Hua K (2021). A time-resolved transcriptome landscape of the developing mouse ovary. Biochem Biophys Res Commun.

[56] Eldani M, Luan Y, Xu PC, Bargar T, Kim S-Y (2020). Continuous treatment with cisplatin induces the oocyte death of primordial follicles without activation. FASEB J.

[57] Li J, Kawamura K, Cheng Y, Liu S, Klein C, Liu S (2010). Activation of dormant ovarian follicles to generate mature eggs. Proc Natl Acad Sci U S A.

[58] He M, Zhang T, Zhu Z, Qin S, Wang H, Zhao L (2020). LSD1 contributes to programmed oocyte death by regulating the transcription of autophagy adaptor SQSTM1/p62. Aging Cell.

[59] Pedersen T (1970). Determination of follicle growth rate in the ovary of the immature mouse. J Reprod Fertil.

[60] Gao M, Zhang T, Chen T, Chen Z, Zhu Z, Wen Y (2024). Polycomb repressive complex 1 modulates granulosa cell proliferation in early folliculogenesis to support female reproduction. Theranostics.

[61] Quinn MCJ, McGregor SB, Stanton JL, Hessian PA, Gillett WR, Green DPL (2006). Purification of granulosa cells from human ovarian follicular fluid using granulosa cell aggregates. Reprod Fertil Dev.

[62] Le HT, Hasegawa Y, Daitoku Y, Kato K, Miznuo-Iijima S, Dinh TTH (2020). Generation of B6-Ddx4^em1(CreERT2)Utr^, a novel CreERT2 knock-in line, for germ cell lineage by CRISPR/Cas9. Genesis.

[63] Dong J, Albertini DF, Nishimori K, Kumar TR, Lu N, Matzuk MM (1996). Growth differentiation factor-9 is required during early ovarian folliculogenesis. Nature.

[64] di Clemente N, Racine C, Pierre A, Taieb J (2021). Anti-Müllerian hormone in female reproduction. Endocr Rev.

[65] Tingen CM, Bristol-Gould SK, Kiesewetter SE, Wellington JT, Shea L, Woodruff TK (2009). Prepubertal primordial follicle loss in mice is not due to classical apoptotic pathways. Biol Reprod.

[66] Ernst EH, Franks S, Hardy K, Villesen P, Lykke-Hartmann K (2018). Granulosa cells from human primordial and primary follicles show differential global gene expression profiles. Hum Reprod.

[67] Spears N, Lopes F, Stefansdottir A, Rossi V, De Felici M, Anderson RA (2019). Ovarian damage from chemotherapy and current approaches to its protection. Hum Reprod Update.

[68] Carmona B, Marinho HS, Matos CL, Nolasco S, Soares H (2023). Tubulin post-translational modifications: the elusive roles of acetylation. Biology (Basel).

[69] Paredes A, Romero C, Dissen GA, DeChiara TM, Reichardt L, Cornea A (2004). TrkB receptors are required for follicular growth and oocyte survival in the mammalian ovary. Dev Biol.

[70] Xu B, Li Z, Li S, Ke H, Zhang Q, Qin Y (2022). Pathogenic variants in TSC2 might cause premature ovarian insufficiency through activated mTOR induced hyperactivation of primordial follicles. Fertil Steril.

[71] Adhikari D, Zheng W, Shen Y, Gorre N, Hämäläinen T, Cooney AJ (2009). Tsc/mTORC1 signaling in oocytes governs the quiescence and activation of primordial follicles. Hum Mol Genet.

[72] Zhang Y, Chen Z, Lin J, Liu J, Lin Y, Li H (2020). The ubiquitin ligase E6AP facilitates HDAC6-mediated deacetylation and degradation of tumor suppressors. Signal Transduct Target Ther.

[73] Shimizu K, Gi M, Suzuki S, North BJ, Watahiki A, Fukumoto S (2021). Interplay between protein acetylation and ubiquitination controls MCL1 protein stability. Cell Rep.

[74] Dai Y, Bo Y, Wang P, Xu X, Singh M, Jia L (2022). Asynchronous embryonic germ cell development leads to a heterogeneity of postnatal ovarian follicle activation and may influence the timing of puberty onset in mice. BMC Biol.

