# Supplementary material for: HDAC6-dependent deacetylation of NGF dictates its ubiquitination and maintains primordial follicle dormancy
Source: Theranostics. 2024 Mar 25;14(6):2345–66. doi: 10.7150/thno.95164 (PMC11024860; doi:10.7150/thno.95164)
Supplement: Supplementary file 1 — Supplementary figures and tables. [file thnov14p2345s1.pdf]

HDAC6-dependent deacetylation of NGF dictates its ubiquitination and maintains primordial follicle dormancy

Supplemental figure legends

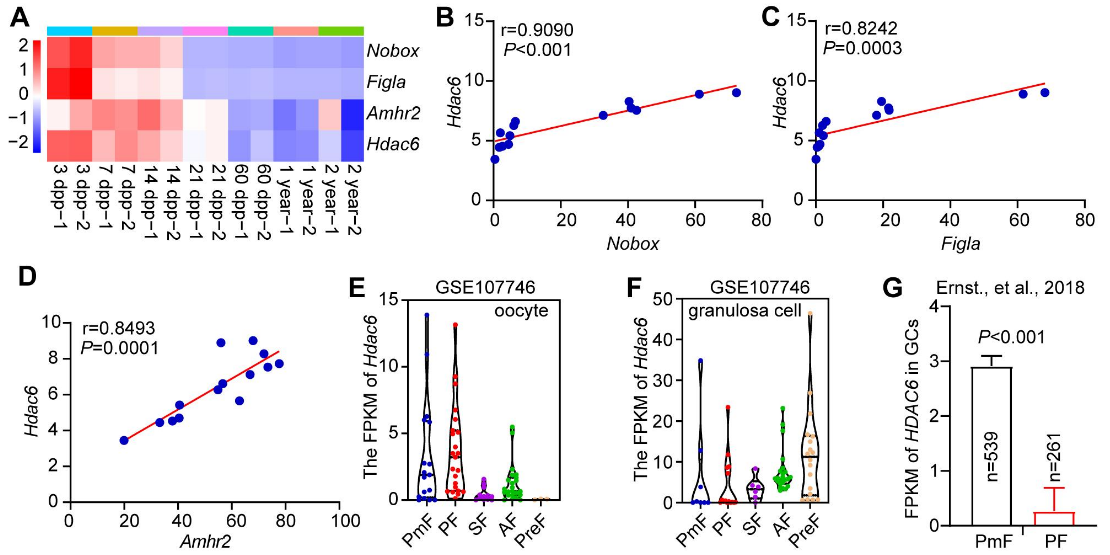

Fig. S1 Expression of *HDAC6* in granulosa cells and oocytes from different developmental stages of mouse and human ovaries

(**A**) Expression of *Hdac6*, *Nobox*, *Figla* and *Amhr2* during mouse ovarian development and aging was analyzed using transcriptome sequencing results from ovarian tissues at different time points (GSE179888). **(B)** Correlation analysis of *Hdac6* and *Nobox* expression during mouse ovarian development and aging (GSE179888). **(C)** Correlation analysis of *Hdac6* and *Figla* expression during mouse ovarian development and aging (GSE179888). **(D)** Correlation analysis of *Hdac6* and *Amhr2* expression during mouse ovarian development and aging (GSE179888). **(E)** Expression of *Hdac6* in oocytes from primordial follicles, primary follicles, secondary follicles, antral follicles and pre-ovulatory follicles was analyzed using human ovarian single-cell transcriptome sequencing data (GSE107746). **(F)** Expression of *Hdac6* in granulosa cells from primordial follicles, primary follicles, secondary follicles, antral follicles and pre-ovulatory follicles was analyzed using human ovarian single-cell transcriptome sequencing data (GSE107746). **(G)** Expression of *Hdac6* in granulosa cells from primordial follicles (PmF) and primary follicles (PF) was analyzed using human ovarian transcriptome sequencing data (Ernst., et al., 2018).

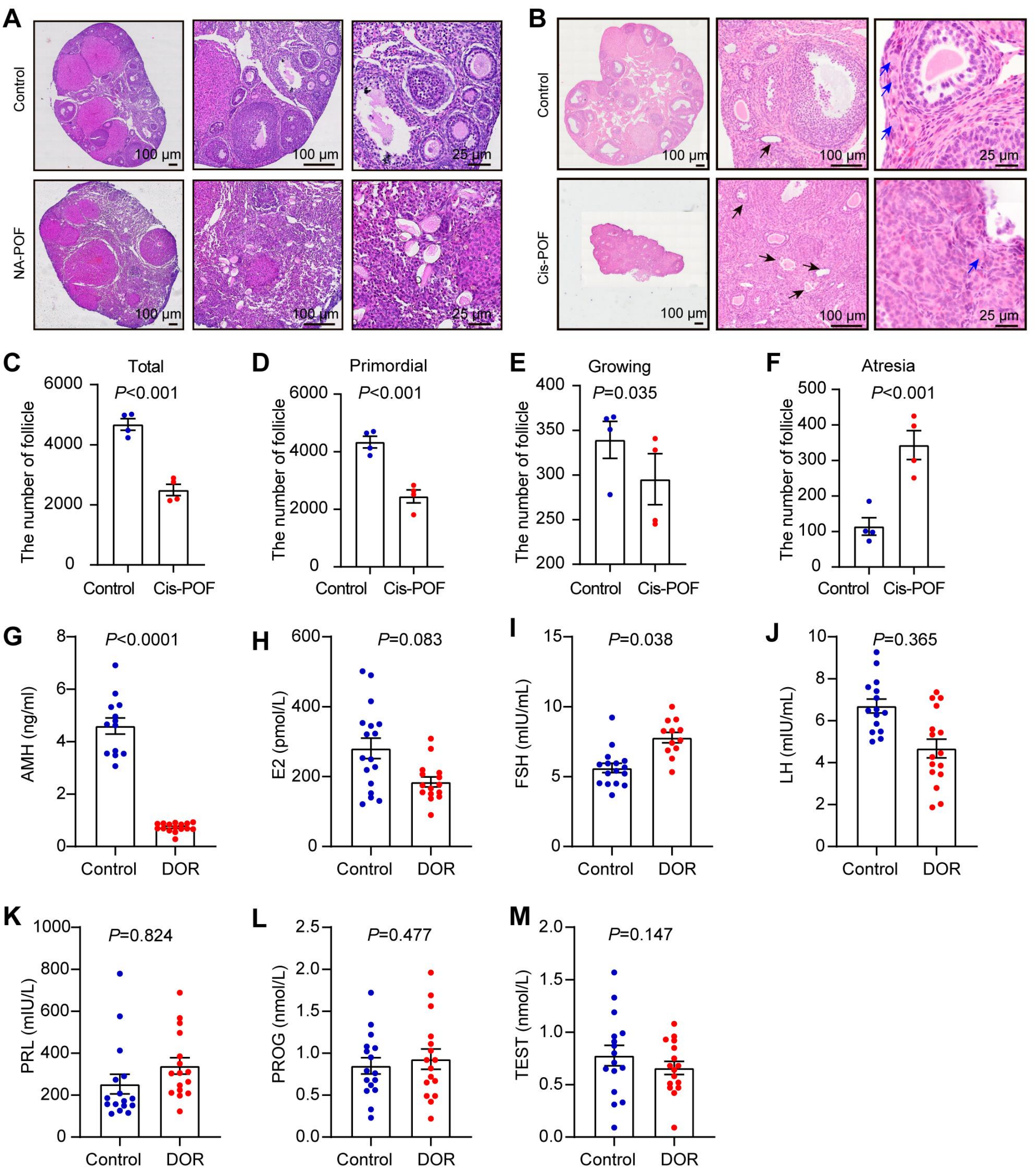

Fig. S2 Validation of the ovarian aging model and the DOR patient

(A) Hematoxylin and eosin staining of ovarian sections from young mouse ovaries and natural aging ovaries. **(B)** Hematoxylin and eosin staining of mouse ovarian sections from young ovaries and cisplatin-induced aging ovaries. Black arrows indicate atresia follicles. Blue arrows indicate primordial follicles. **(C)** Number of total follicles in the whole ovary. **(D)** Number of primordial follicles in the whole ovary. **(E)** Number of growing follicles in the whole ovary. **(F)** Number of atresia follicles in the whole ovary. **(G)** AMH levels in serum of DOR patients. **(H)** E2 levels in serum of DOR patients. **(I)** FSH levels in serum of DOR patients. **(J)** LH levels in serum of DOR patients. **(K)** PRL levels in serum of DOR patients. **(L)** PROG levels in serum of DOR patients. **(M)** TEST levels in serum of DOR patients.

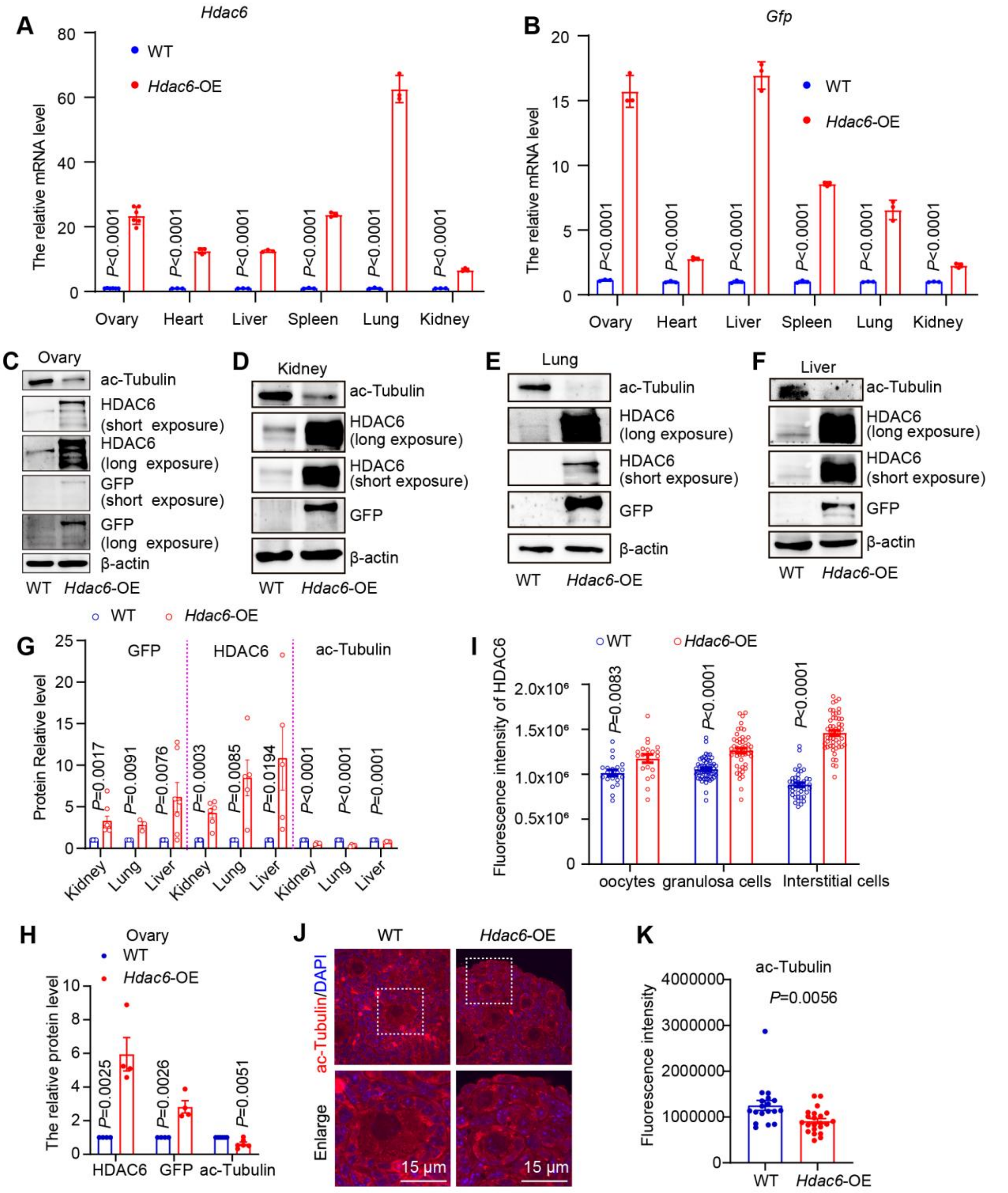

Fig. S3 Overexpression efficiency assay from different organs in *Hdac6*-OE mouse

(**A**) The relative mRNA level of *Hdac6* in the ovary, heart, liver, spleen, lung and kidney. **(B)** The relative mRNA level of *Gfp* in the ovary, heart, liver, spleen, lung and kidney. **(C-F)** The western blot of HDAC6, GFP and ac-Tubulin in *Hdac6*-OE mouse ovary (C), kidney (D), lung (E) and liver (F). **(G-H)** The statistical analysis of figure C-F. **(I)** Statistical analysis of HDAC6 fluorescence intensity in *Hdac6*-OE mouse ovarian oocyte, granulosa cells and interstitial cells. **(J)** Immunofluorescence of ac-α-Tubulin in the ovaries from 7 dpp *Hdac6*-OE transgenic mice. Ac-α-Tubulin is labeled red. The nuclei were stained with DAPI (blue). Scale bars, 15 μm. **(K)** The statistical analysis of figure J.

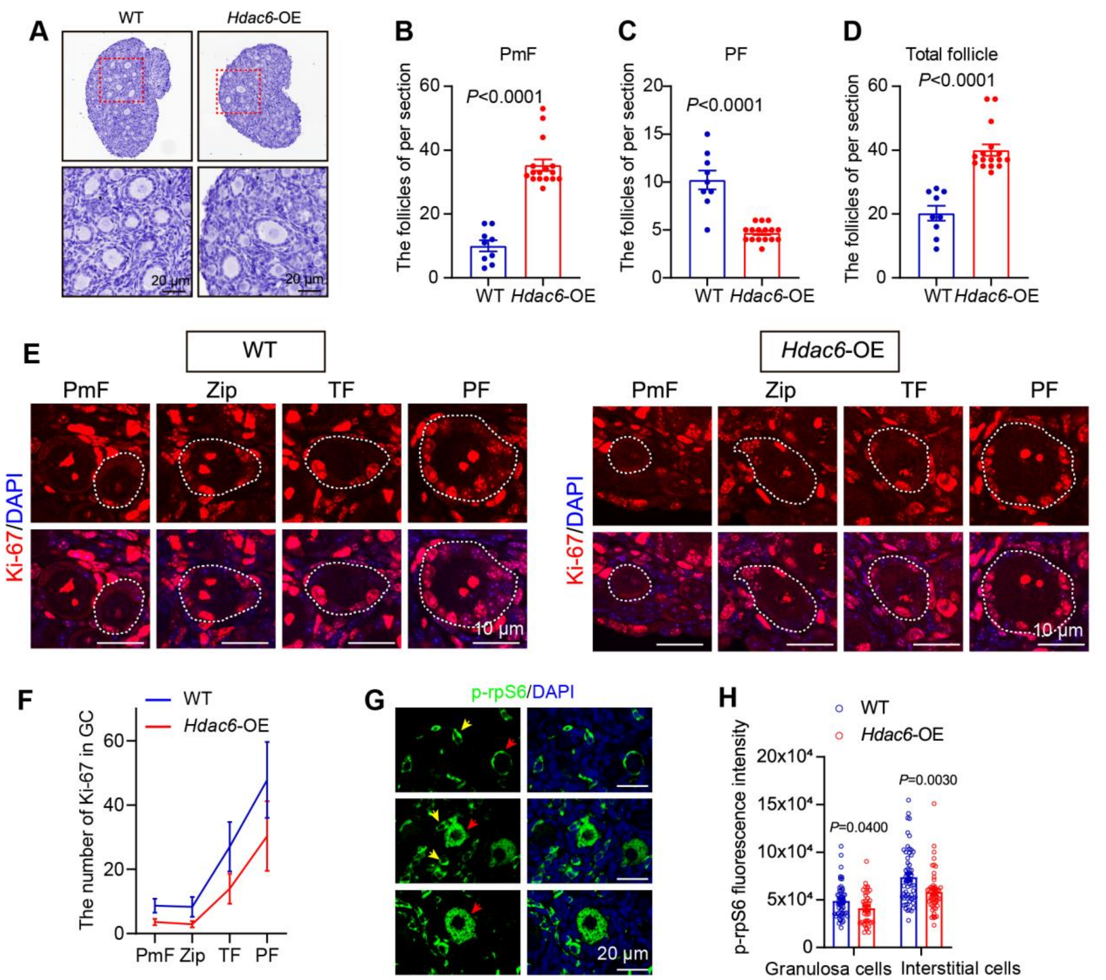

Fig. S4 Overexpression of *Hdac6* increases follicular reserve and delays primordial follicle activation

(A) Hematoxylin staining of ovary sections from 7 dpp *Hdac6*-OE mice. **(B-D)** The number of PmF, PF and total follicle in per section. **(E)** The rate of change to Ki67-positive granulosa cells in PmF, Zip, TF and PF. **(F)** Immunofluorescence of p-rpS6 from 7 dpp mouse ovaries. p-rpS6 is labeled green. The nuclei were stained with DAPI (blue). Scale bars, 20 μm. **(G)** Statistical analysis of p-rpS6 fluorescence intensity in *Hdac6*-OE mouse ovarian oocyte, granulosa cells and interstitial cells.

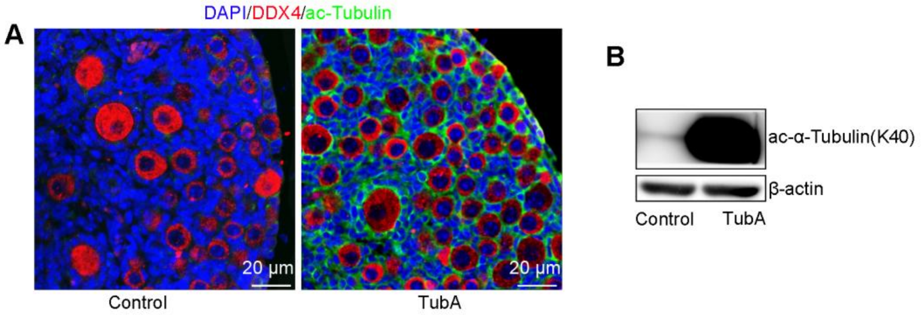

Fig. S5 The protein level of ac-Tubulin was increased in TubA-treated mouse ovaries

(A) Immunofluorescence of ac-Tubulin from TubA-treated mouse ovaries. The 2 dpp ovaries were cultured with TubA for 2 days. Ac-α-Tubulin is labeled green. DDX4 is labeled red. The nuclei were stained with DAPI (blue). Scale bars, 20 μm. **(B)** The protein level of ac-α-Tubulin from TubA-treated mouse ovaries. The 2 dpp ovaries were cultured with TubA for 2 days.

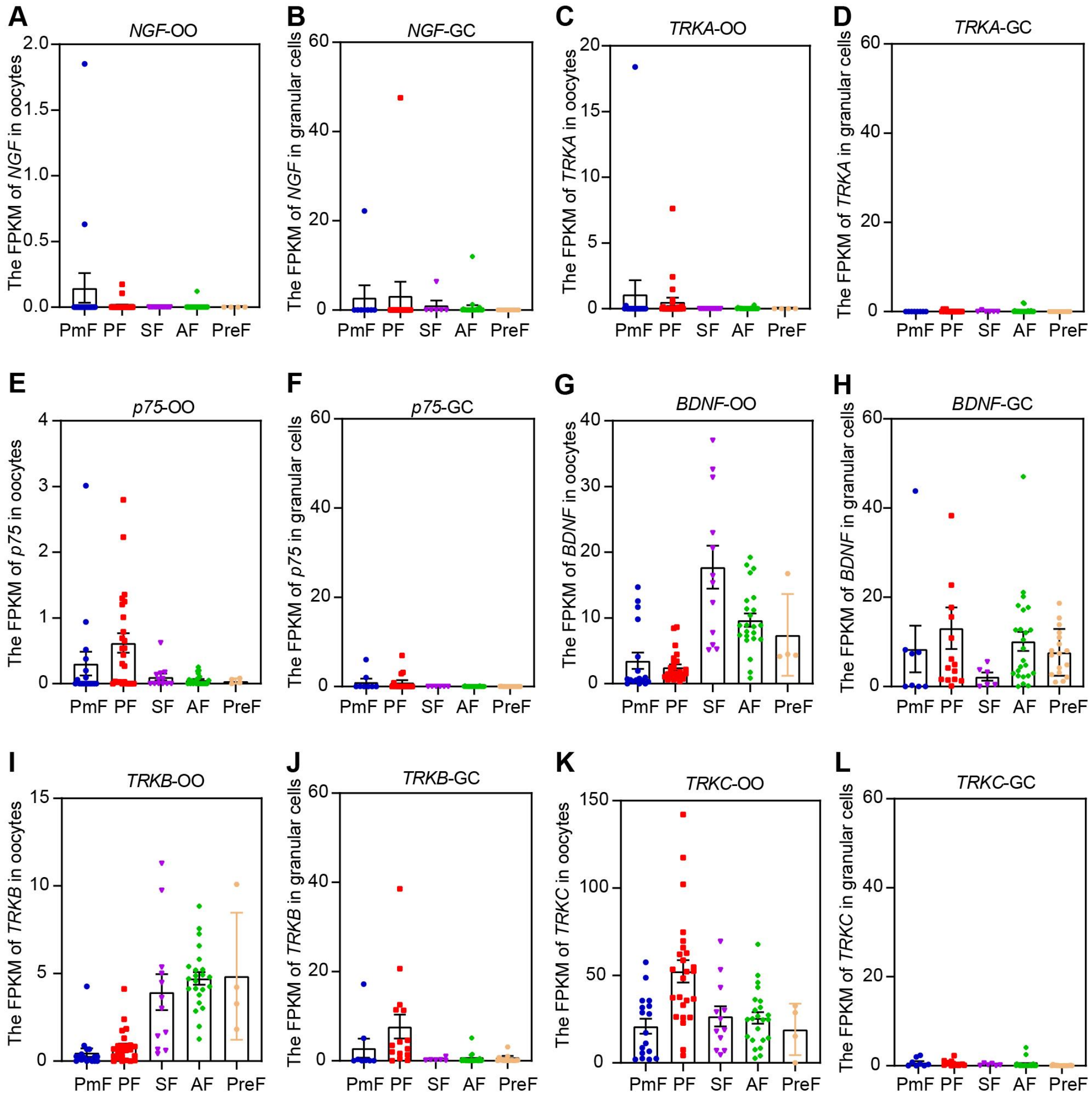

Fig. S6 The FPKM values of key molecules of neural ligand/receptor signaling pathways in granulosa cells and oocytes of human follicles at different stages of follicular development.

(A-B) The FPKM values of *NGF* in oocytes and granulosa cells from different stages of human follicle. **(C-D)** The FPKM values of *TRKA* in oocytes and granulosa cells from different stages of human follicle. **(E-F)** The FPKM values of *p75* in oocytes and granulosa cells from different stages of human follicle. **(G-H)** The FPKM values of *BDNF* in oocytes and granulosa cells from different stages of human follicle. **(I-J)** The FPKM values of *TRKB* in oocytes and granulosa cells from different stages of human follicle. **(K-L)** The FPKM values of *TRKC* in oocytes and granulosa cells from different stages of human follicle.

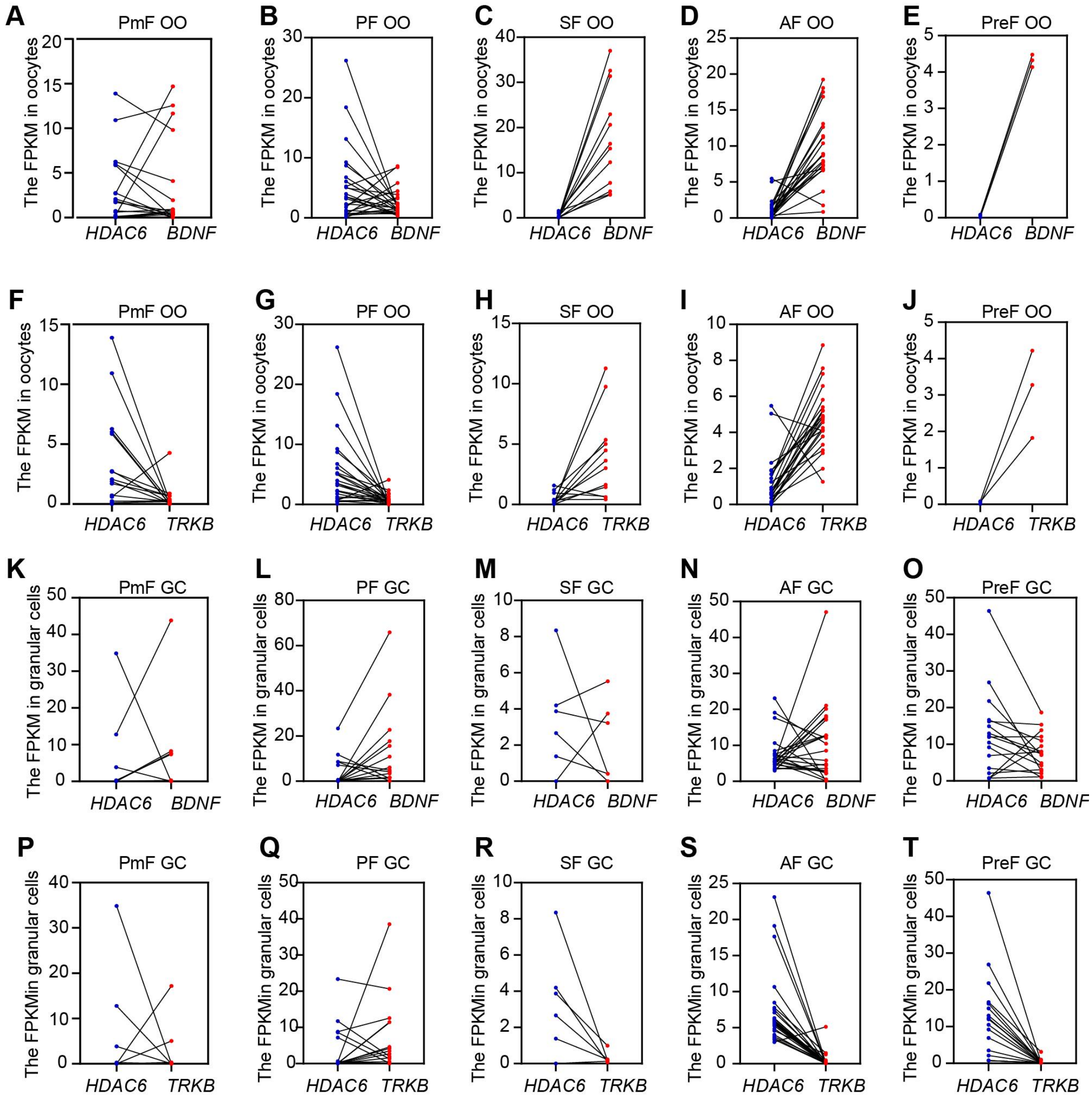

Fig. S7 Expression levels of HDAC6 and BDNF/TRKB are quantified in human different stage follicle

(A-E) Expression levels of HDAC6 and BDNF were quantified in the same oocyte from primordial follicle, primary follicle, secondary follicle, antral follicle and pre-ovulatory follicle. **(F-J)** Expression levels of HDAC6 and TRKB were quantified in the same oocyte from primordial follicle, primary follicle, secondary follicle, antral follicle and pre-ovulatory follicle. **(K-O)** Expression levels of HDAC6 and BDNF were quantified in the same granulosa cell from primordial follicle, primary follicle, secondary follicle, antral follicle and pre-ovulatory follicle. **(P-T)** Expression levels of HDAC6 and TRKB were quantified in the same granulosa cell from primordial follicle, primary follicle, secondary follicle, antral follicle and pre-ovulatory follicle.

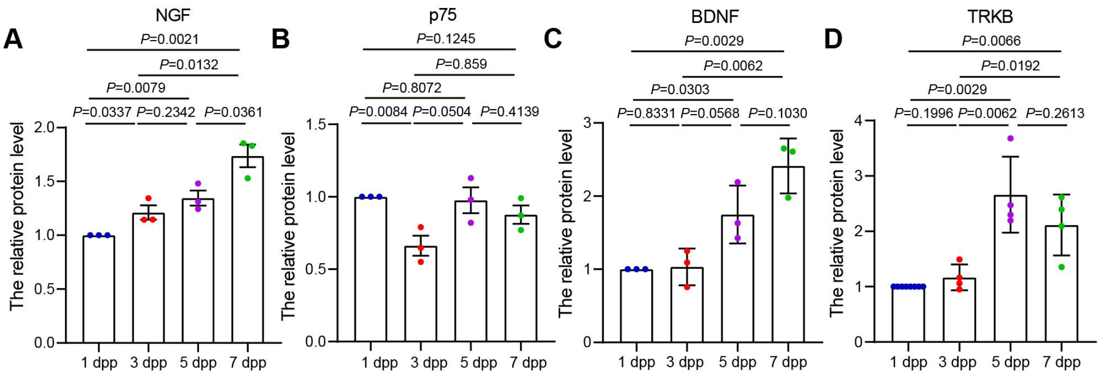

Fig. S8 The relative protein level of NGF, TRKA, p75, BDNF and TRKB under physiological mouse ovaries.

(A-D) The statistical analysis on relative protein level of NGF, p75, BDNF and TRKB from 1 dpp, 3 dpp, 5 dpp to 7 dpp newborn mouse ovaries.

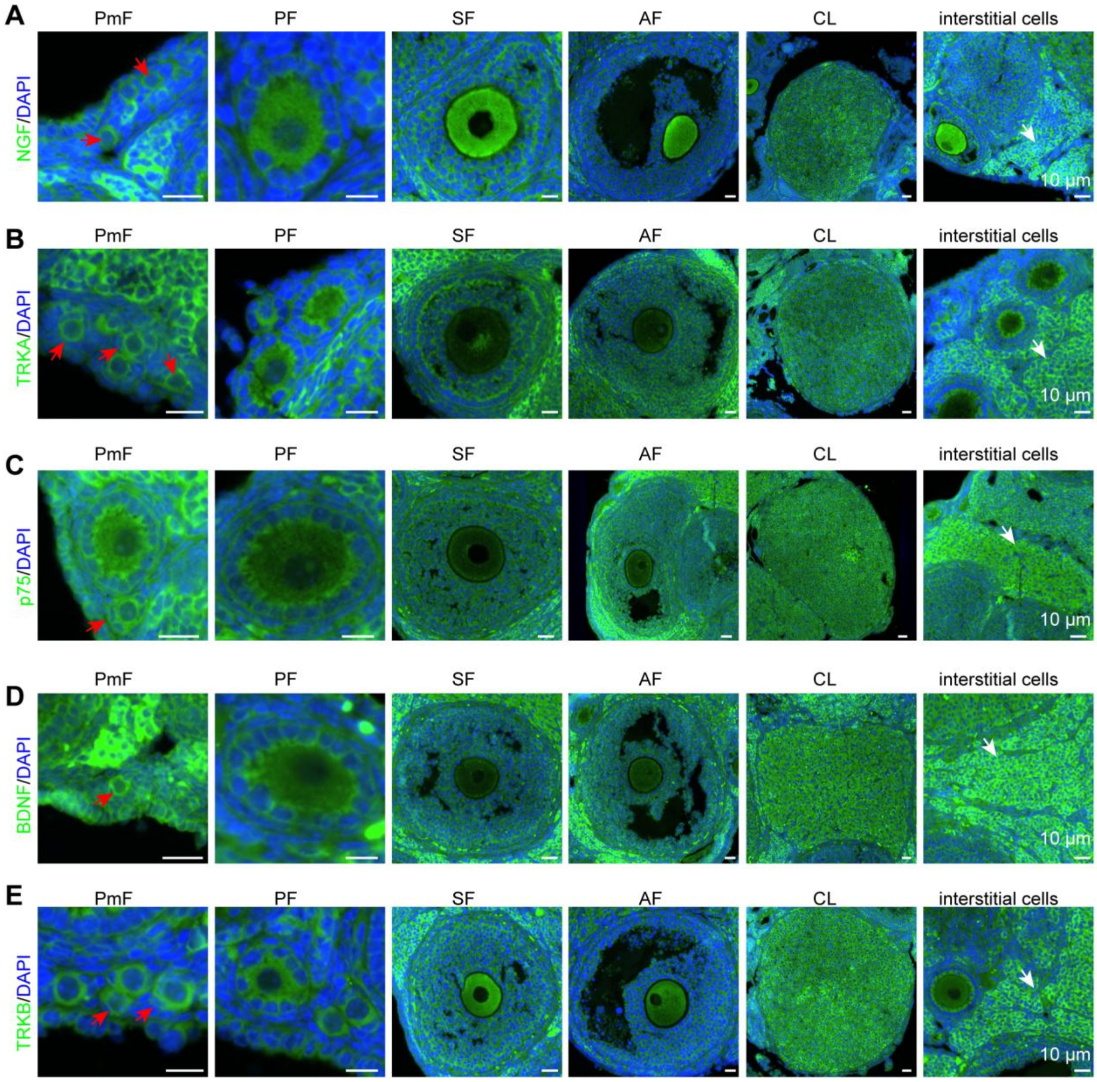

Fig. S9 The subcellular localization of NGF, TRKA, p75, BDNF and TRKB in the adult mouse ovary.

(A-E) The immunofluorescence of NGF, TRKA, p75, BDNF and TRKB were examined by immunofluorescence in adult mouse ovarian sections, respectively. NGF, TRKA, p75, BDNF and TRKB was labeled green, respectively. The nucleus was stained by DAPI (blue). Red arrows indicate primordial follicles. White arrows indicate interstitial cells. Scale bars, 10 μm.

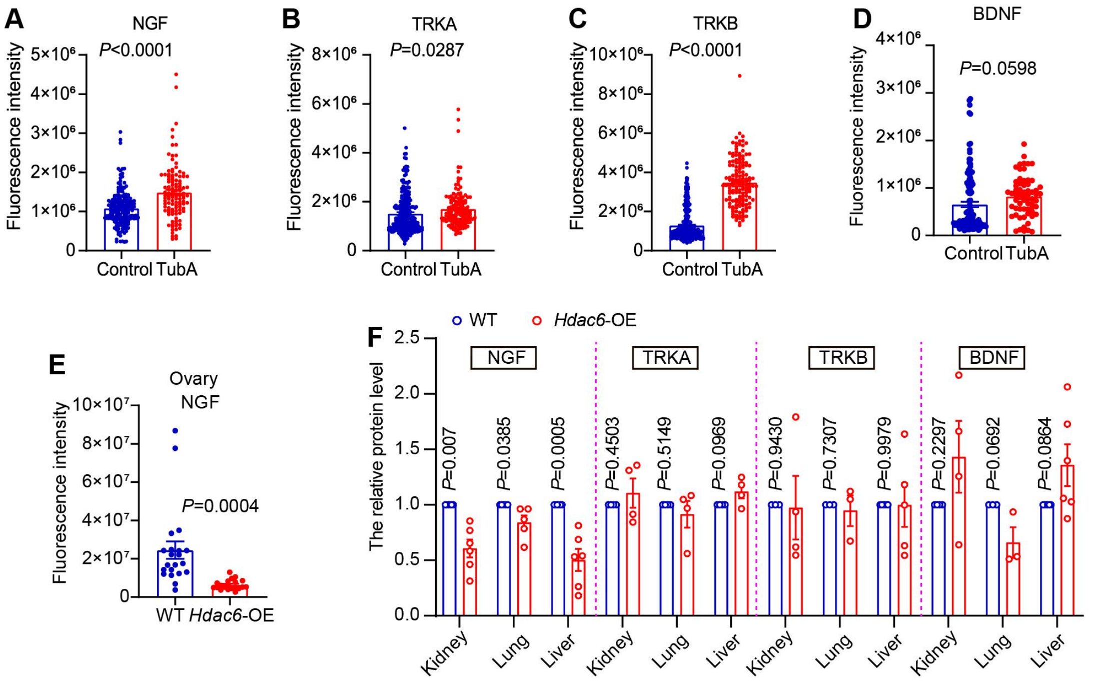

Fig. S10 The relative expression level of NGF, TRKA, p75, BDNF and TRKB

(A-D) The statistical analysis on fluorescence intensity of NGF, TRKA, TRKB and BDNF form TubA treated KGN cells, respectively. The KGN cells were cultured with or without TubA for 24 hours. **(E)** The statistical analysis on fluorescence intensity of NGF form 7 dpp *Hdac6*-OE mouse ovaries. **(F)** The statistical analysis on the relative protein level of NGF, TRKA, BDNF and TRKB from 7 dpp *Hdac6*-OE mouse kidney, lung and liver, respectively.

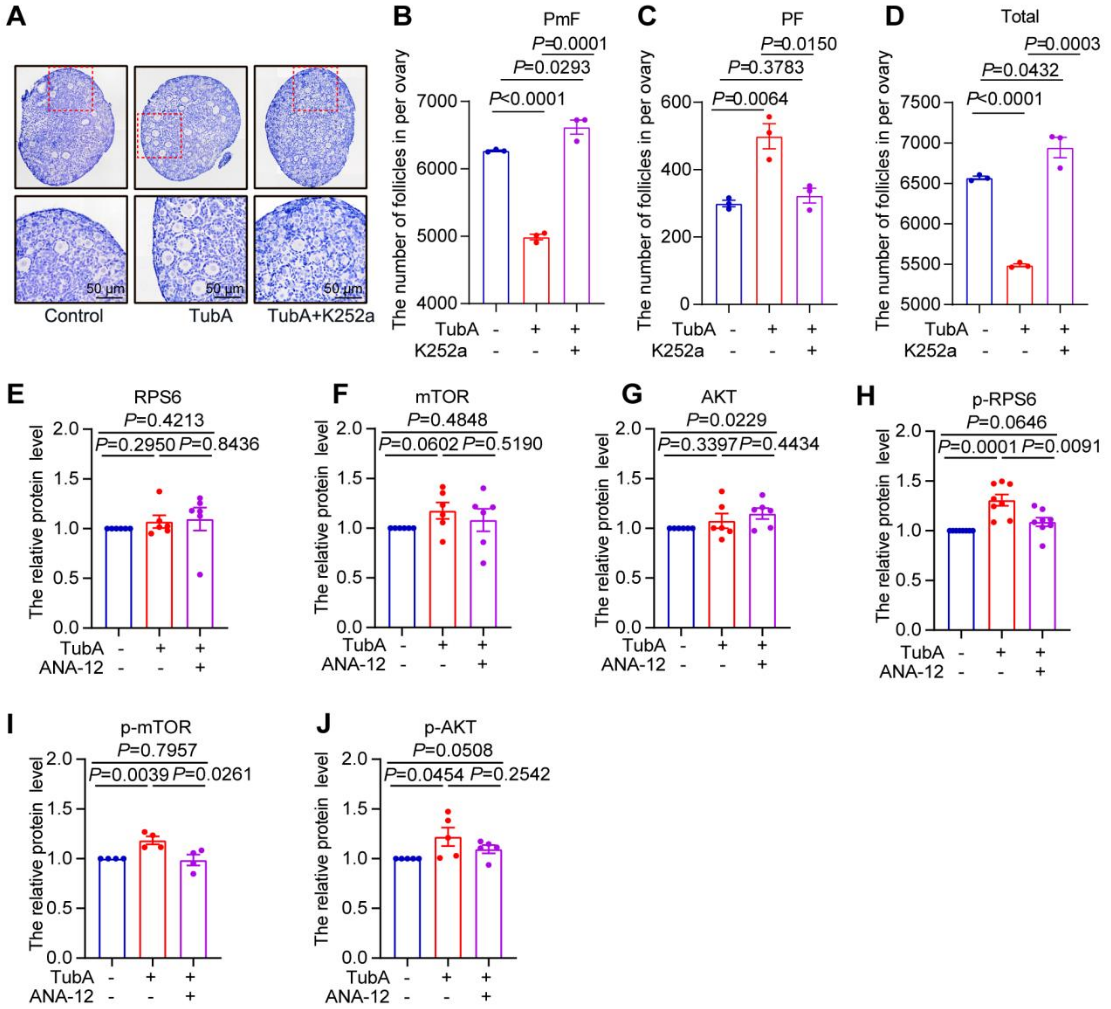

Fig. S11 HDAC6 regulates mouse primordial follicle activation via neuroligands ligand /receptor signaling pathway

(A) Hematoxylin staining of the ovarian section with TubA and K252a treated ovary. The 2 dpp ovaries were cultured for 3 days. **(B-D)** The whole ovary follicle counts about primordial follicle, primary follicle and total follicle. **(E-J)** The statistical analysis on the relative protein level of RPS6, mTOR, AKT, p-RPS6, p-mTOR and p-AKT from TubA and ANA-12 treated mouse ovaries, respectively. The 2 dpp ovaries were cultured with TubA and ANA-12 for 2 days.

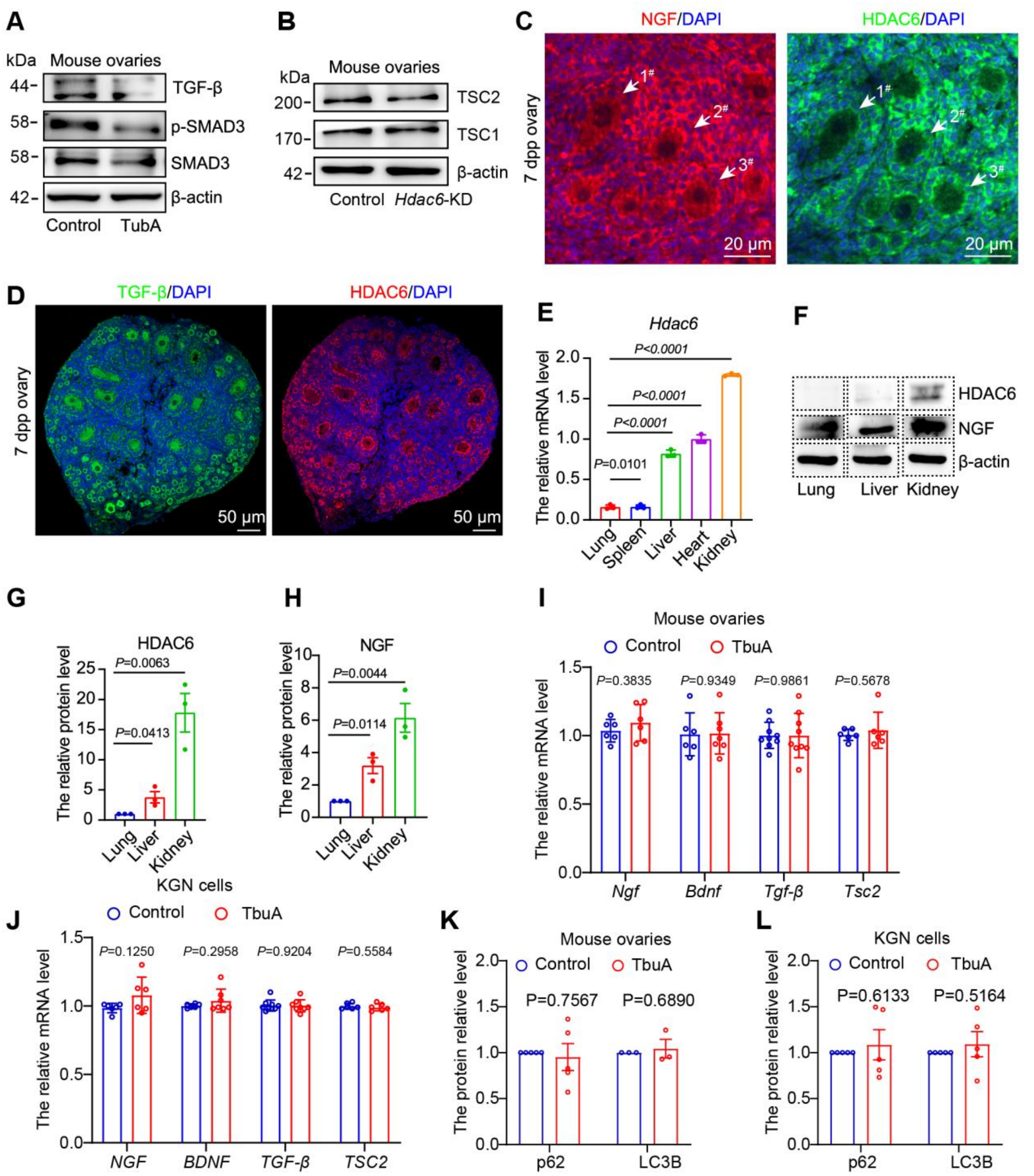

Fig. S12 Autophagy levels were not changed in TubA-treated mouse ovaries and KGN cells

(A) The western blot of TGF-β, SMAD and p-SMAD3 in TubA treated-mouse ovaries. 3 dpp mouse ovaries were cultured with or without TubA for 2 days. **(B)** The western blot of TSC1 and TSC2 in *Hdac6*-KD mouse ovaries. **(C)** HDAC6 and NGF were co-located in granulosa cells of follicles. The 7 dpp mouse ovaries' adjacent sections were stained for NGF (red) or HDAC6 (green). The nucleus was stained by DAPI (blue). Scale bars, 50 μm. **(D)** HDAC6 and TGF-β were co-located in granulosa cells of follicles. The 7 dpp mouse ovaries' adjacent sections were stained for TGF-β (green) or HDAC6 (red). The nucleus was stained by DAPI (blue). Scale bars, 50 μm. **(E)** The mRNA level of *Hdac6* in wild-type mouse lung, spleen, liver, heart and kidney. **(F-H)** The protein level of HDAC6 in wild-type mouse lung, liver and

kidney. **(I)** The relative mRNA level of *Ngf*, *Bdnf, Tgf-β* and *Tsc2 in* TubA-treated ovaries. 3 dpp mouse ovaries were cultured with or without TubA for 2 days. *β-actin* was used as an internal control. **(J)** The relative mRNA level of *NGF*, *BDNF, TGF-β* and *TSC2 in* TubA treated KGN cells. KGN cells were cultured with or without TubA for 24 hours. *β-ACTIN* was used as an internal control. **(K-L)** The statistical analysis on relative protein level of LC3B and p62 from TubA-treated mouse ovaries and KGN cells, respectively.

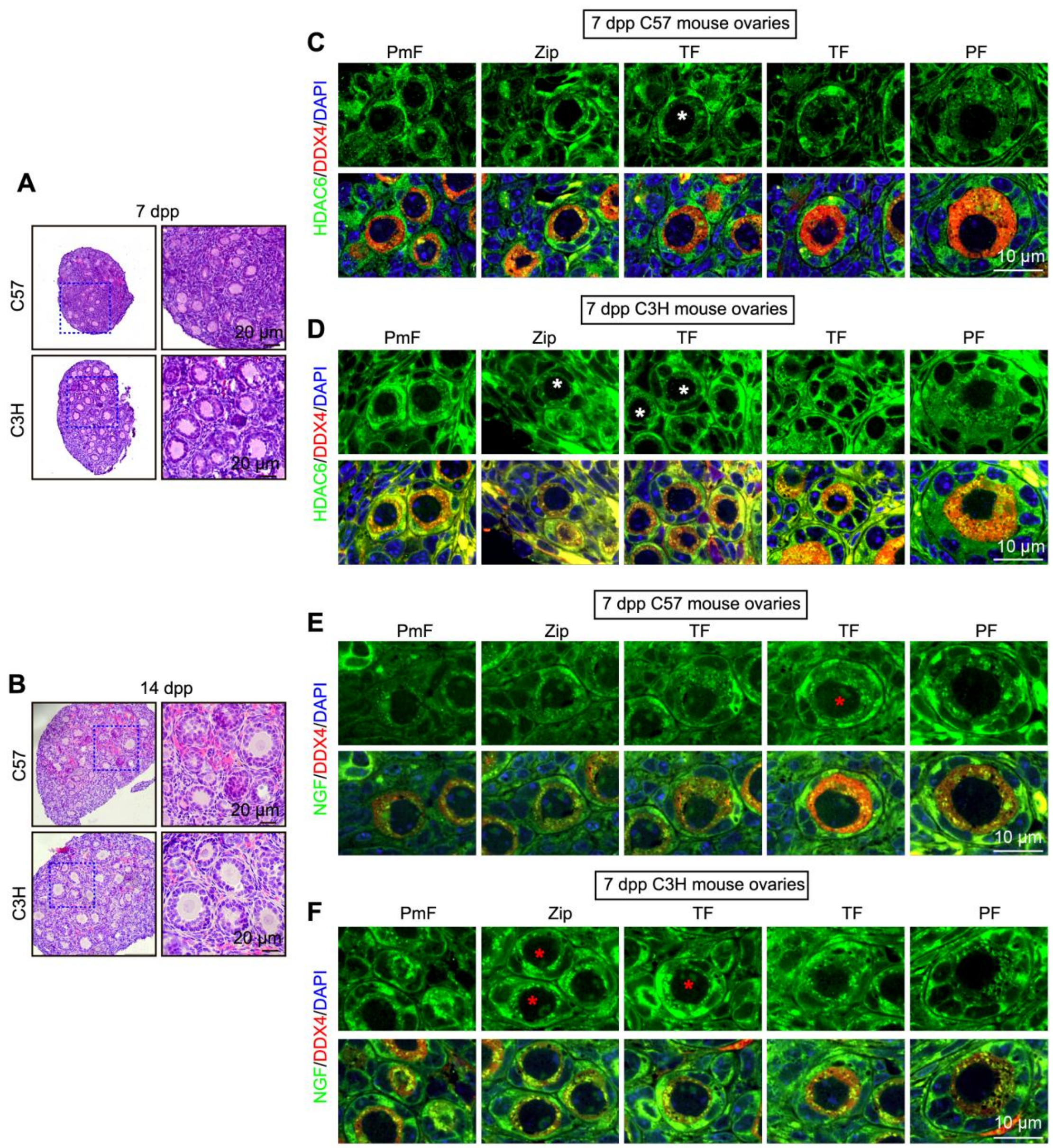

Fig. S13 The expression of HDAC6 and NGF in newborn C57 and C3H mouse ovaries
(A) The hematoxylin eosin stain of 7 dpp C57 and C3H mouse ovaries. **(B)** The hematoxylin eosin stain of 14 dpp C57 and C3H mouse ovaries. **(C-D)** The expressioin of HDAC6 was examined by immunofluorescence in 7 dpp C57 and C3H mouse ovaries, respectively. These HDAC6 signals (green) were co-stained with DDX4 (red). DDX4 is the marker of oocyte. The nucleus was stained by DAPI (blue). White asterisks indicate primordial follicles with

low expression of HDAC6. Scale bars, 10 μm. **(E-F)** The expression of NGF was examined by immunofluorescence in 7 dpp C57 and C3H mouse ovaries, respectively. These NGF signals (green) were costained with DDX4 (red). DDX4 is the marker of oocyte. The nucleus was stained by DAPI (blue). Red asterisks indicate primordial follicles with high expression of NGF. Scale bars, 10 μm.

Supplemental Tables

Table S1 Antibodies

Antibodies	Vendors; Cat. No.	Source	Dilution/Applications
HDAC6	Beyotime Biotechnology; AF7071	Rabbit	1:1000 (WB); 1:200 (IF)
HDAC6	Cell Signaling Technology; #7612	Rabbit	1:200 (IP)
TSC1	Cell Signaling Technology; #6935	Rabbit	1:1000 (WB)
SMAD3	Cell Signaling Technology; #9523	Rabbit	1:1000 (WB)
p-SMAD3	Cell Signaling Technology; #9520	Rabbit	1:1000 (WB)
Ubiquitin (P37)	Cell Signaling Technology; #58395	Rabbit	1:1000 (WB)
Normal Rabbit IgG	Cell Signaling Technology; #2729	Rabbit	1:200 (IP)
Mouse IgG	Proteintech; B900620	Mouse	1:200 (IP)
NGF	Beyotime Biotechnology; AF1411	Rabbit	1:1000 (WB); 1:200 (IF);1:50 (IP)
p75 NGF	Beyotime Biotechnology; AF1033	Rabbit	1:500 (WB); 1:200 (IF)
TrkA	Beyotime Biotechnology; AF1630	Rabbit	1:400 (WB); 1:200 (IF)
TrkB	SERVICEBIO;GB11295-1-100	Rabbit	1:400 (WB); 1:200 (IF)
BDNF	Bioworld Technology; BS6533	Rabbit	1:500 (WB); 1:200 (IF)
AKT	Cell Signaling Technology; #4691	Rabbit	1:1000 (WB)
p-AKT	Cell Signaling Technology; #4060	Rabbit	1:1000 (WB)
FoxO3a	Cell Signaling Technology; #12829	Rabbit	1:400 (IF)
mTOR	Cell Signaling Technology; #2983	Rabbit	1:1000 (WB)
p-mTOR	Cell Signaling Technology; #5536	Rabbit	1:1000 (WB)
RPS6	Beyotime Biotechnology; AF7917	Rabbit	1:1000 (WB)
p-RPS6	Beyotime Biotechnology; AF5917	Rabbit	1:1000 (WB); 1:200 (IF)
P62	Abcam; ab56416	Mouse	1:1000 (WB)
LC3B	Beyotime Biotechnology; AF5225	Rabbit	1:500 (WB)
TSC2	Proteintech; 68380-1-Ig	Mouse	1:2000 (WB)
TGF Beta 1	Proteintech; 21898-1-AP	Rabbit	1:2000 (WB); 1:400 (IF)
TGF-β 1/2	Beyotime Biotechnology; AF0297	Rabbit	1:1000 (WB)

GFP tag	Proteintech; 50430-2-AP	Rabbit	1:1000 (WB); 1:150 (IP)
Acetyl-α-Tubulin (Lys40) (D20G3) XP®	Cell Signaling Technology;#5335	Rabbit	1:1000 (WB); 1:800 (IF)
Acetyl-lysine	Cell Signaling Technology;#9441	Rabbit	1:100 (IP)
DDX4/MVH	Abcam; Ab27591	Mouse	1:200 (IF)
Ki-67	SERVICEBIO; GB111499	Rabbit	1:200 (IF)
β-actin	Cwbiotech; CW0096M	Mouse	1:3000 (WB)
FITC	Proteintech; SA00003-8	Donkey	1:200 (IF)
Alexa Fluor® 594	YEASEN; 34112ES60	Donkey	1:200 (IF)
DAPI	Merck; D9542		1:1000 (IF)

Table S2 Primers real-time PCR

Primer	Sequence	Application
Hdac6-F-Mouse	*TCCACCGGCCAAGATTCTTC*	qPCR
Hdac6-R-Mouse	*CAGCACACTTCTTTCCACCAC*	qPCR
Ngf-F-Mouse	*CCAGTGAAATTAGGCTCCCTG*	qPCR
Ngf-R-Mouse	*CCTTGGCAAAACCTTTATTGGG*	qPCR
β-actin-F-Mouse	*GTGACGTTGACATCCGTAAAGA*	qPCR
β-actin-R-Mouse	*GCCGGACTCATCGTACTCC*	qPCR
Bdnf-F-Mouse	*TCATACTTCGGTTGCATGAAGG*	qPCR
Bdnf-R-Mouse	*AGACCTCTCGAACCTGCCC*	qPCR
Gfp-F-Mouse	*GACAAGCAGAAGAACGGCATCA*	qPCR
Gfp-R-Mouse	*TCCAGCAGGACCATGTGAT*	qPCR
Tsc2-F-Mouse	*TAGAACAAGCAATGGATCTGGTG*	qPCR
Tsc2-R-Mouse	*GCTGAGGAGACATTCGGCTG*	qPCR
Tgf-β1-F-Mouse	*TGACGTCACTGGAGTTGTACGG*	qPCR
Tgf-β1-R-Mouse	*GGTTCATGTCATGGATGGTGC*	qPCR
HDAC6-F-HUMAN	*GTTTGAGAAAGGGGCTGCG*	qPCR
HDAC6-R-HUMAN	*GGTTCTGCCTACTTCTTCGCT*	qPCR
β-ACTIN-F-HUMAN	*CATGTACGTTGCTATCCAGGC*	qPCR
β-ACTIN-R-HUMAN	*CTCCTTAATGTCACGCACGAT*	qPCR
TGF-β1-F-HUMAN	*CAACACATCAGAGCTCCGAGA*	qPCR
TGF-β1-R-HUMAN	*GAGCCTCAGCAGACGCAG*	qPCR
NGF-F-HUMAN	*GGGAGCGCAGCGAGTTT*	qPCR
NGF-R-HUMAN	*TGCCGATCAGAAAAGCTGTG*	qPCR
BDNF-F-HUMAN	*CAATAGCCCCCATGCTCTGT*	qPCR
BDNF-R-HUMAN	*CCTTGTCCTCGGATGTTTGC*	qPCR
TSC2-F-HUMAN	*ATAGCTGTTACCTCGACGAGT*	qPCR
TSC2-R-HUMAN	*TGCAGGGAGACCTCTATGTCC*	qPCR
Hdac6-OE-mutant-F	*TGAGCAAAGACCCCAACGAGAAG*	Genetype

Hdac6-OE-mutant-R	*CTTTATTAGCCAGAAGTCAGATGC*	Genetype
Hdac6-OE-wt-F	*CACTTGCTCTCCCAAAGTCGCTC*	Genetype
Hdac6-OE-wt-R	*ATACTCCGAGGCGGATCACAA*	Genetype
